# Cultivation of Dominant Freshwater Bacterioplankton Lineages Using a High-Throughput Dilution-to-Extinction Culturing Approach Over a 1-Year Period

**DOI:** 10.3389/fmicb.2021.700637

**Published:** 2021-07-27

**Authors:** Suhyun Kim, Md. Rashedul Islam, Ilnam Kang, Jang-Cheon Cho

**Affiliations:** ^1^Department of Biological Sciences and Bioengineering, Inha University, Incheon, South Korea; ^2^Bacteriophage Biology Laboratory, Guelph Research and Development Centre, Agriculture and Agri-Food Canada, Guelph, ON, Canada; ^3^Department of Biological Sciences, Center for Molecular and Cell Biology, Inha University, Incheon, South Korea

**Keywords:** freshwater, bacterioplankton, cultivation, bacterial community, dilution-to-extinction, high-throughput culturing

## Abstract

Although many culture-independent molecular analyses have elucidated a great diversity of freshwater bacterioplankton, the ecophysiological characteristics of several abundant freshwater bacterial groups are largely unknown due to the scarcity of cultured representatives. Therefore, a high-throughput dilution-to-extinction culturing (HTC) approach was implemented herein to enable the culture of these bacterioplankton lineages using water samples collected at various seasons and depths from Lake Soyang, an oligotrophic reservoir located in South Korea. Some predominant freshwater bacteria have been isolated from Lake Soyang via HTC (e.g., the acI lineage); however, large-scale HTC studies encompassing different seasons and water depths have not been documented yet. In this HTC approach, bacterial growth was detected in 14% of 5,376 inoculated wells. Further, phylogenetic analyses of 16S rRNA genes from a total of 605 putatively axenic bacterial cultures indicated that the HTC isolates were largely composed of *Actinobacteria, Bacteroidetes, Alphaproteobacteria, Betaproteobacteria, Gammaproteobacteria*, and *Verrucomicrobia*. Importantly, the isolates were distributed across diverse taxa including phylogenetic lineages that are widely known cosmopolitan and representative freshwater bacterial groups such as the acI, acIV, LD28, FukuN57, MNG9, and TRA3–20 lineages. However, some abundant bacterial groups including the LD12 lineage, *Chloroflexi*, and *Acidobacteria* could not be domesticated. Among the 71 taxonomic groups in the HTC isolates, representative strains of 47 groups could either form colonies on agar plates or be revived from frozen glycerol stocks. Additionally, season and water depth significantly affected bacterial community structure, as demonstrated by 16S rRNA gene amplicon sequencing analyses. Therefore, our study successfully implemented a dilution-to-extinction cultivation strategy to cultivate previously uncultured or underrepresented freshwater bacterial groups, thus expanding the basis for future multi-omic studies.

## Introduction

Driven by the recent development of next- and third-generation sequencing technologies, myriads of single-cell amplified genomes and metagenome-assembled genomes have been retrieved from diverse environments using multi-omics approaches (Zhang et al., 2010). These innovative culture-independent approaches have enabled the discovery of genomic information from uncultured bacteria and archaea, which resulted in the creation of the “microbial dark matter” concept (Rinke et al., 2013; Williams and Embley, 2014). Further, these approaches have provided crucial insights into the diversity and function of underrepresented microbial communities. Although many efforts have been continuously made to cultivate the uncultured (Lewis et al., 2021), the development of cultivation approaches for uncultured microbes is lagging behind, as the majority of naturally occurring microbial assemblages are not readily cultivable via conventional cultivation approaches based on agar plates.

Among the various known cultivation approaches, high-throughput dilution-to-extinction culturing (HTC) has been successfully applied to oligotrophic marine ecosystems by inoculating microbial cells into a physically separated microwell with natural seawater-based media (Connon and Giovannoni, 2002; Cho and Giovannoni, 2004; Salcher et al., 2015; Kim et al., 2017). Using HTC, many abundant marine bacterial clades such as SAR11 (“*Candidatus* Pelagibacter”) (Rappe et al., 2002; Song et al., 2009), SAR 116 (“*Candidatus* Puniceispirillum”) (Henson et al., 2016; Lee et al., 2019), OM43 (Giovannoni et al., 2008), SAR92 (Stingl et al., 2007), OM60 (Cho et al., 2007), SUP05 (Shah et al., 2017; Spietz et al., 2019) and others have been isolated and their growth and genome properties have been further studied. The success of HTC has been attributed to the physical isolation of microorganisms in low-volume growth chambers (microtiter wells) enriched with specific microorganisms under culture conditions mimicking those of their natural habitats (e.g., natural seawater medium).

However, although HTC has allowed for the cultivation of several previously uncultured major marine bacterial lineages, far fewer studies have focused on the culture of freshwater bacteria. Bacterial groups that are abundant in freshwater lakes worldwide belong to diverse lineages of *Alphaproteobacteria*, *Betaproteobacteria*, *Actinobacteria*, *Bacteroidetes*, *Planctomycetes*, *Chloroflexi*, and *Verrucomicrobia* (Glockner et al., 2000; Zwart et al., 2002; Newton et al., 2011). Among abundant freshwater bacterial groups, the genera *Polynucleobacter* and *Limnohabitans* of the class *Betaproteobacteria* are relatively easier to grow than other dominant microbial groups (Hahn, 2003; Kasalický et al., 2010) and thus have been frequently isolated using a filtration-acclimatization method coupled with standard agar plating methods. Therefore, these two genera are among the most widely studied freshwater bacterial groups (Boscaro et al., 2013; Hahn et al., 2016a; Sangwan et al., 2016; Jezberova et al., 2017). In fact, the successful domestication of non-colony-forming abundant freshwater bacterial lineages was only recently achieved using HTC. To name a few examples, this approach has allowed for the cultivation of the lineages LD28 (“*Candidatus* Methylopumilus”), a freshwater methylotrophic *Betaproteobacteria* (Salcher et al., 2015, 2019); LD12 (“*Candidatus* Fonsibacter”), a freshwater sister group of the marine SAR11 clade (Henson et al., 2018); and acI (“*Candidatus* Nanopelagicales”), the predominant actinobacterial lineage found in most freshwater lakes (Kang et al., 2017; Neuenschwander et al., 2018; Kim et al., 2019).

Our research group has performed HTC experiments since 2014 in Lake Soyang, the largest freshwater reservoir in Korea. Although many abundant freshwater bacterial lineages have been isolated from Lake Soyang during the HTC campaigns, our research has mainly focused on the stable cultivation of acI bacterial cultures, as the initial HTC cultures of acI bacteria were not revived from frozen glycerol stocks. We have also reported the complete genome sequences of four acI strains (Kang et al., 2017), as well as the growth characteristics of two acI strains that were successfully maintained in freshwater-based media supplemented with catalase (Kim et al., 2019, 2020). Moreover, our group also characterized the genomes of bacteriophages that infect bacterial strains belonging to the LD28 clade and members of the family *Comamonadaceae*, which were isolated from our HTC trials (Moon et al., 2017, 2018). However, large-scale HTC studies encompassing different seasons and water depths in Lake Soyang have not yet been reported. Therefore, our study sought to obtain phylogenetic snapshots of bacterial isolates cultured via HTC, as well as to characterize the culture-independent bacterial community structure of water samples collected from Lake Soyang at two depths and four seasons. Among a total of 5,376 inoculated microtiter wells, 605 putative axenic bacterial cultures were isolated and were found to belong to as-yet-uncultured groups of *Actinobacteria, Bacteroidetes, Verrucomicrobia, Alphaproteobacteria, Betaproteobacteria*, and *Gammaproteobacteria*.

## Materials and Methods

### Study Site, Sampling, and Physicochemical Parameters

Lake Soyang is a deep, large, and oligotrophic-to-mesotrophic artificial reservoir located in South Korea. The maximum depth of the reservoir is 118 m (the deepest in Korea) and water overturn begins in early winter and lasts until the surface water warms again in spring. Therefore, Lake Soyang is classified as a typical warm monomictic lake. In Korea, heavy rainfall occurs during the monsoon season (July–August) and flows into the reservoir’s metalimnion (Kim and Kim, 2006). Therefore, phytoplankton densities reach their maximum after the monsoon season, as nutrients are gradually dispersed from the metalimnion to the epilimnion and water temperature increases (Kim et al., 2000; Jung et al., 2016). Considering the temporal and spatial changes in water characteristics of Lake Soyang, water samples were collected from 1 to 50 m depths at one location in front of a dam (37°57’11′′ N, 127°49’02′′ E) using a Niskin sampler from April 2014 to January 2015 (Supplementary Figure 1 and Supplementary Table 1). Sampling was conducted four times at 3-month intervals during this period, each designated “spring” (14 April 2014), “summer” (16 July 2014), “autumn” (17 October 2014), and “winter” (15 January 2015). Samples collected at 1 m and 50 m depths were labeled “surface” and “bottom,” respectively. The water samples collected were immediately stored in an icebox cooler and transported to the laboratory within 8 h.

The temperature, pH, dissolved oxygen, conductivity, and salinity of each water sample were measured *in situ* with a YSI 556 MPS Multiprobe System (YSI Incorporated, Yellow Springs, OH, United States). Concentrations of ammonium, nitrite, nitrate, phosphate, silicate, total dissolved nitrogen, and dissolved organic carbon were determined by the National Instrumentation Center for Environmental Management (NICEM), Korea, following the Standard Methods for Examination of Water and Wastewater guidelines (APHA, 2005). After passing the 1 L water samples through a GF/F filter (Pall, NY, United States) and extracting the resulting material with 90% acetone, chlorophyll-*a* concentration was measured spectrofluorometrically with a Turner Designs^TM^ 10-AU fluorometer (Turner Designs, Sunnyvale, CA).

### Isolation of Bacterial Strains Using HTC

A total of eight HTC campaigns were conducted with the samples collected in spring, summer, autumn, and winter. Supplementary Figure 2 illustrates the overall experimental scheme. HTC growth media was prepared as described in previous studies (Kang et al., 2017; Kim et al., 2020) using the same water samples for each inoculum. The 1-L lake water samples were passed through a 0.2 μm filter, autoclaved at 121°C for 1.5 h, sparged with air for 3 h, and enriched with the ingredients (nitrogen, phosphorous, carbohydrates, amino acids, trace metals, and vitamins) listed in Supplementary Table 2. After counting the total 4′,6-diamidino-2-phenylinole (DAPI)-stained microbial cells using an epifluorescence microscope (Nikon 80i, Nikon, Japan), either 2 or 5 cells per 1 ml of culture medium were inoculated into 48-well microtiter plates (BD Falcon, Franklin Lakes, NJ, United States). The plates were incubated at 15°C in a walk-in cold room in the dark for 1 month. Next, 100-μl samples of the cultures were stained with a 5 × final concentration of SYBR Green I (Invitrogen, United States), after which microbial growth in the plates was screened via flow cytometry (Guava EasyCyte Plus, Millipore, MA, United States). Wells with ≥ 10^5^ cells/ml were recorded as growth-positive, after which 400 μl of these growth-positive cultures were cryopreserved in a glycerol suspension (10%, v/v) at −80°C. Of the remaining 500 μl of culture, 100 μl was transferred to a microcentrifuge tube to serve as PCR template, and 10 μl was spotted onto Reasoner’s 2A (R2A) (Reasoner and Geldreich, 1985) and 1/10R2A (BD Difco, United States) agar plates to determine colony-forming activity. At this stage, culture IDs prefixed with IMCC (Inha Microbe Culture Collection) were assigned to each growth-positive culture.

### 16S rRNA Gene Sequencing of HTC Isolates and Phylogenetic Analyses

Growth-positive culture samples were freeze-thawed three times in microcentrifuge tubes and used directly as PCR templates using the 27F-B and 1492R universal bacterial primers (Lane, 1991) and a thermal cycler (PTC 200, Bio-Rad, United States). The PCR products were then analyzed via Sanger sequencing using the 800R primer (5′-TAC CAG GGT ATC TAA TCC-3′) by Macrogen Inc. (Seoul, Korea). The 16S rRNA gene sequences were quality-checked and trimmed with the BioEdit software version 7.2.5 (Hall, 1999). Only unambiguously base-called sequences were linked to putatively pure cultures and used for downstream phylogenetic analyses. Taxonomic assignment of 16S rRNA genes from pure cultures was performed using the classification algorithm implemented in the Mothur software (Schloss et al., 2009) using the SILVA (SSU Ref NR 123) (Quast et al., 2013) and ExTaxon-e databases (Kim et al., 2012). For further phylogenetic analysis, the sequences were aligned using the SINA web aligner (Pruesse et al., 2012), imported into the ARB software (Ludwig et al., 2004) equipped with the SILVA database, and inserted into a guide tree generated based on the freshwater bacterial classification scheme proposed by Newton et al. (2011). The aligned 16S rRNA gene sequences of the HTC isolates and other reference sequences were exported and used to infer maximum-likelihood trees using RAxML (version 8.1.17) with rapid bootstrapping (a maximum of 1,000 bootstrap replicates). The generated maximum-likelihood trees were imported again into the ARB database and manually curated for phylogenetic grouping according to the aforementioned freshwater bacterial classification scheme (Newton et al., 2011). To classify the most abundant actinobacterial acI and acIV lineages in more detail, further tribe-based phylogenetic analyses were performed using the freshwater-specific FreshTrain database (Rohwer et al., 2018).

### Colony-Forming Activity and Recovery From Cryopreservation of the HTC Isolates

The colony-forming activity of liquid HTC cultures was tested by spotting 10 μl of culture samples on R2A and 1/10R2A plates (BD Difco, Warwick, RI, United States). The inoculated plates were then incubated at 15°C for 1 month and visually inspected to assess colony formation. Using the colonies formed on the agar plates, 16S rRNA gene sequences were determined via the bead-beating method and the sequences were compared with those of the initial liquid-cultured HTC isolates. Once the identity of the colonies was confirmed, additional sequences were obtained using the 518F primer (5′-CCA GCA GCC GCG GTA ATA CG-3′) and assembled with the sequences obtained with the 800R primer. Colonies were also cryopreserved as 10% glycerol suspensions at −80°C.

For the HTC isolates that did not form colonies on R2A or 1/10R2A, HTC-isolated glycerol stocks were used to test whether cryopreserved isolates could resuscitate during the freeze-thawing process. At least one representative strain from each phylogenetic group (i.e., tribes such as acI-A1) represented in the maximum-likelihood trees was selected and retrieved from the glycerol stocks. Cryopreserved stocks were thawed at room temperature, after which 200 μl of the stocks were inoculated into 20 ml of fresh media with the same composition as the media from the initial HTC experiments. Cellular growth during incubation at 15°C was monitored via flow cytometry every week and isolates with a 10-fold increase in cell numbers relative to their initial cell densities were considered “reviving-positive.” Bacterial strains whose growth was not observed after 4 weeks of incubation or exhibited a less than 10-fold increase were considered “reviving-negative.” Finally, upon confirming that the 16S rRNA gene sequences of the revived cultures matched those of the initial HTC isolates, additional sequences were obtained using the 518F primer as described above.

### 16S rRNA Gene Pyrosequencing

To analyze the bacterial community diversity in Lake Soyang, 1 L of eight lake water samples was filtered through a 0.2 μm-pore-size 47 mm polyethersulfone membrane filter (Pall, NY, United States) after prefiltration with a 3.0 μm-pore-size membrane filter. After cell lysis by lysozyme treatment on membrane filters, DNA was extracted using the DNeasy Blood and Tissue Kit (Qiagen, Hilden, Germany) according to the manufacturer’s instructions. The V1-V3 region of the 16S rRNA genes was amplified using fusion primers, whose design was based on that of the 27F and 519R universal primers. Pyrosequencing was performed by ChunLab, Inc. (Seoul, Korea) using a Roche 454 GS Junior sequencer.

Raw sequencing reads were processed with the Mothur program following the 454 SOP protocol (Schloss et al., 2011). Supplementary Figure 3 details the amplicon sequence processing procedure and the number of sequencing reads screened at each step. Upon processing a total of 132,625 raw sequencing reads, 63,954 high-quality reads (14,231 unique sequences) were used for taxonomic profiling. Taxonomic assignment of unique sequence reads was made based on Mothur’s classification tool with a bootstrap cut-off score of 80 using the SILVA database (SSU Ref NR 123) as a reference. The aligned sequence reads were used to generate an uncorrected pairwise distance matrix. The number of sequences was normalized prior to statistical analysis by randomly resampling reads of each sample to the same size based on the sample with the smallest sampling size (*n* = 6,266 sequences in this study). Operational taxonomic unit (OTU) clustering was conducted using an average-neighbor algorithm at a 98% similarity cutoff, then normalized for further statistical analysis. Distance calculation by the Bray-Curtis method, principal coordinate analysis (PCoA), and hierarchical clustering using the unweighted pair-group method with arithmetic means (UPGMA) was conducted using the vegan package in R version 3.3.2 (Dixon, 2003). Additionally, HTC isolate sequences (queries) and high-quality unique pyrosequencing reads (database) were compared using a local BLAST (NCBI BLASTN 2.2.25+) (Altschul et al., 1997) to compare cultured bacterial diversity and bacterial community structure. This sequence comparison was performed for > 300 bp sequences based on a 97 and 98.7% sequence similarity cut-off.

### Nucleotide Sequence Accession Numbers

The 16S rRNA gene sequences of the HTC isolates were deposited in the GenBank/EMBL/DDBJ databases under accession numbers MW884569–MW885173. The accession numbers for each sample are also listed in Supplementary Data. The pyrosequencing data reported herein were also submitted to the Sequence Read Archive (SRA) database under accession number SRR14278392–SRR14278399.

## Results and Discussion

### Overview of HTC Results

A total of eight HTC experiments were conducted using the water samples collected from Lake Soyang at 1 and 50 m depths in spring (April 2014), summer (July 2014), autumn (October 2014), and winter (January 2015). The studied lake exhibited a thermocline in spring and summer (Supplementary Figure 1), and physicochemical parameters and total prokaryotic numbers varied with season and depth during the HTC campaigns (Supplementary Table 1). The culture medium was prepared immediately prior to the HTC experiments at each sampling point to reflect the variations in water chemistry; however, incubation temperatures were maintained at 15°C regardless of sampling point to render optimal bacterial growth rates. Bacterial growth (≥ 10^5^ cells/ml) was detected in 14.0% (750/5,376) of the inoculated wells (Table 1). The culturing efficiency (i.e., the number of positive wells divided by the number of inoculated wells) was highest in winter (22.9%; 308/1,344 wells), followed by spring (19.0%), autumn (9.1%), and summer (4.8%). The culturing efficiencies of the surface waters (1 m; 34.2%; 459/1,344 wells) were higher than those of the bottom waters (50 m; 21.7%; 291/1,344 wells), except for the summer samples. Among the eight HTC experiments, the highest number of growth positives was isolated in spring from the surface water sample (27.8%; 187 out of 672 wells), whereas the lowest numbers were obtained from the surface water sample in summer (1.5%; 10 out of 672 wells). Culturability, which was calculated using Button’s formula (Button et al., 1993), showed a similar pattern to the culturing efficiency, with high culturability values in spring and winter coupled with low values in the summer surface samples (Table 1). It is not clear why the summer surface water exhibited low culturability; however, we speculate that this may be due to high levels of UV irradiation and the formation of reactive oxygen species (Häder et al., 2015).

**TABLE 1:** Summary of high-throughput dilution-to-extinction culturing experiments conducted in this study.

			No. of wells							
Sampling depth (m)	No. of cells inoculated (cells/well)	No. of wells inoculated in each season	Growth-positives^a^/Pure cultures^b^/Colony-formers^c^				Culturability (*V*)^d^			
			Spring	Summer	Autumn	Winter	Spring	Summer	Autumn	Winter
1	5	192	81/66/14	5/5/5	51/40/11	69/54/10	0.110	0.005	0.062	0.089
	2	480	106/86/12	5/5/4	50/40/3	92/77/9	0.125	0.005	0.055	0.107
50	5	192	32/25/2	22/18/5	10/6/2	61/57/13	0.037	0.024	0.011	0.077
	2	480	36/24/4	33/24/10	11/9/1	86/69/14	0.039	0.036	0.012	0.099
Total		5,376^*e*^	750/605/119				−			

*^a^Wells with cell densities of 1.0 × 10^5^ cells/ml or higher. ^b^Determined by electropherogram of 16S rRNA gene sequences. ^c^Colony forming bacteria on 1/10R2A and R2A. ^d^V = −ln(1−p)/X, where p is the proportion of growth-positives and X is the number of inoculated cells (Button et al., 1993). ^e^Total number of wells inoculated throughout the entire HTC experiment (1,344 wells × 4 seasons).*

**FIGURE 1:**
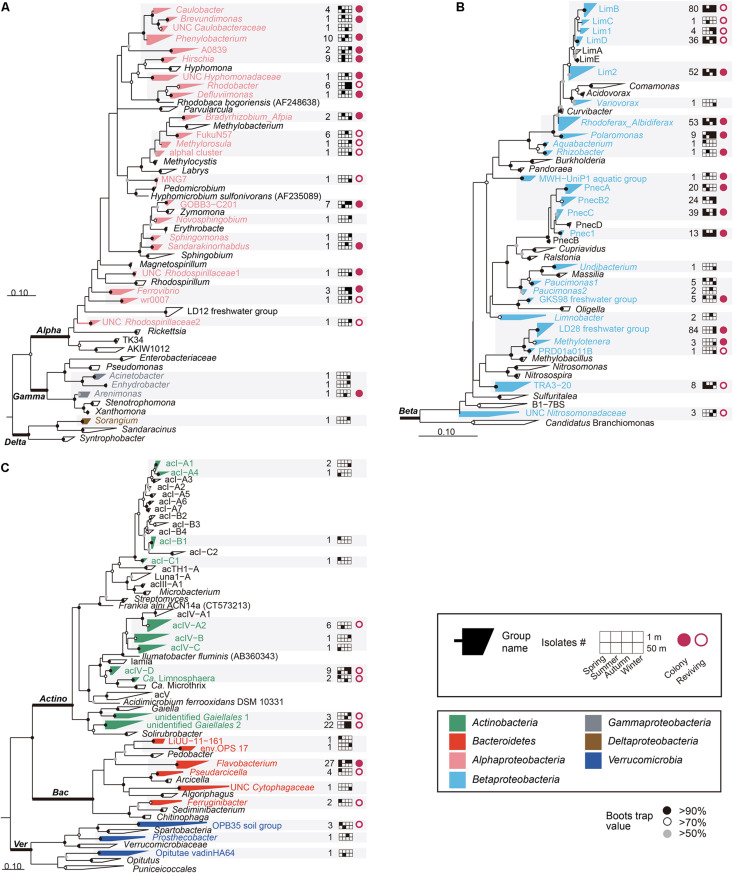
Maximum-likelihood phylogenetic trees based on 16S rRNA gene sequences showing the positions of the cultivated isolates within **(A)** *Alphaproteobacteria, Gammaproteobacteria*, and *Deltaproteobacteria*; **(B)** *Betaproteobacteria*; **(C)** *Actinobacteria, Bacteroidetes*, and *Verrucomicrobia*. Bacterial/group names are listed at the end of the tree tips. The phylogenetic groups represented by the HTC strains isolated in this study are displayed in different colors depending on the different phyla/classes, along with the number of isolates, information on seasons and depths, and colony-forming and reviving capability. UNC: uncultured. Bootstrap supporting values are shown at the nodes as filled solid circles (>90%), empty circles (>70%), and gray circles (>50%).

PCR amplification and sequencing of 16S rRNA genes from 750 wells that contained ≥ 10^5^ cells/ml elucidated the presence of 605 putatively pure cultures and 145 mixed-cultures, and therefore only the axenic cultures were used for further phylogenetic analyses (Table 1). Among the 605 putatively pure cultures, only 119 isolates formed colonies on R2A and/or 1/10R2A. Most of the colony-forming bacteria belonged to the phylum *Proteobacteria*, whereas none of the HTC isolates belonging to the phylum *Actinobacteria* formed colonies (Figure 1). Nonetheless, it is worth noting that the colony-forming ability of the HTC isolates might have been underestimated, as their growth was only tested in R2A-based media and colony-forming ability varies greatly in a medium-dependent fashion.

**FIGURE 2:**
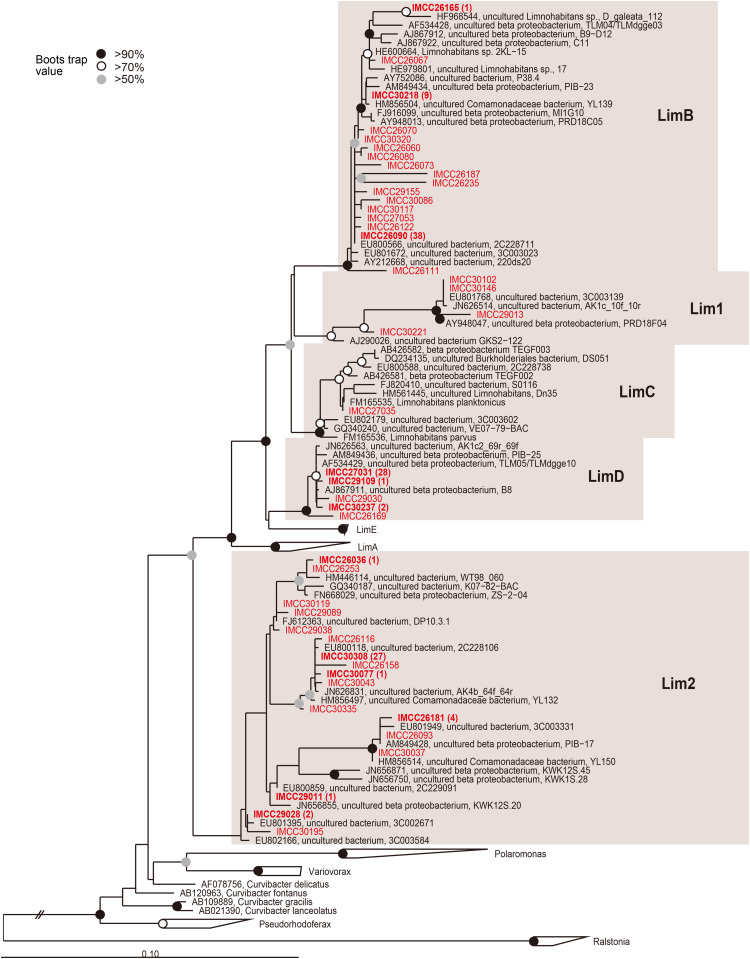
Maximum-likelihood phylogenetic tree based on 16S rRNA gene sequences showing the positions of the HTC isolates within the *Limnohabitans* lineage. The HTC isolates are prefixed with the “IMCC” abbreviation (Inha Microbe Culture Collection) and marked in red. HTC isolates sharing identical 16S rRNA gene sequences are indicated by one representative strain and the numbers of the other isolates sharing the same sequences are indicated in parentheses. The Lim1 and Lim2 linages were named in this study. Bootstrap supporting values are shown at the nodes as solid circles (>90%), empty circles (>70%), and gray circles (>50%).

**FIGURE 3:**
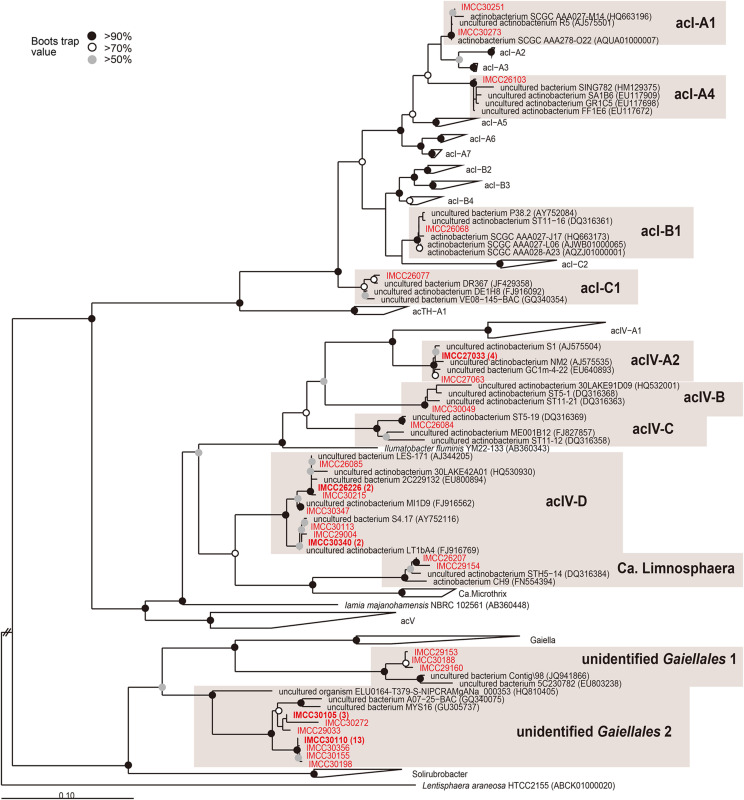
Maximum-likelihood phylogenetic tree based on 16S rRNA gene sequences showing the positions of the HTC isolates within the phylum *Actinobacteria*. The HTC isolates are prefixed with the “IMCC” abbreviation (Inha Microbe Culture Collection) and marked in red. HTC isolates sharing identical 16S rRNA gene sequences are indicated by one representative strain and the numbers of the other isolates sharing the same sequences are indicated in parentheses. The “unidentified *Gaiellales* 1” and “unidentified *Gaiellales* 2” lineages were named in this study. Bootstrap supporting values are shown at the nodes as solid circles (>90%), empty circles (>70%), and gray circles (>50%).

After obtaining partial 16S rRNA gene sequences from all HTC isolates, as well as almost-full sequences of colony-forming isolates or revived HTC isolates from frozen stocks, taxonomic assignment of the HTC isolates was conducted using the EzTaxon-e, SILVA classification scheme in the Mothur software, and FreshTrain database (Supplementary Data and Supplementary Table 3). The 16S rRNA gene sequence comparison using the databases indicated that the 605 putatively pure cultures identified herein belonged to the phyla *Actinobacteria* (49 strains), *Bacteroidetes* (36 strains), and *Verrucomicrobia* (5 strains) and the classes *Alphaproteobacteria* (62 strains), *Betaproteobacteria* (449 strains), *Gammaproteobacteria* (3 strains), and *Deltaproteobacteria* (1 strain). Maximum-likelihood trees generated using the sequences from these 605 pure cultures and their relatives indicated that the HTC isolates obtained in this study were affiliated with diverse phylogenetic clades comprising different lineages, tribes, or genera (Figure 1). The HTC isolates were classified into 71 groups (i.e., acI-A1 and LimB), based on the classification scheme proposed by Newton et al. (2011) and SILVA taxonomy (version 123) (Figure 1, Supplementary Data, and Supplementary Table 3). Figure 1 illustrates the overall phylogenetic classifications of the isolates and provides detailed information on the isolation sources (season and depth), colony-forming capability, and revivability from glycerol stocks. Detailed phylogenetic trees for specific phylogenetic groups are presented in Figures 2, 3 and Supplementary Figures 4–6.

### Phyla/Classes Represented by the Bacterial Isolates Obtained From HTC

### *Alphaproteobacteria, Gammaproteobacteria*, and *Deltaproteobacteria*

Out of a total of 605 HTC isolates, 515 bacterial strains (85.1%) were assigned to the phylum *Proteobacteria*. At the class level, the isolates were found to belong to the *Alphaproteobacteria* (62 strains), *Betaproteobacteria* (449 strains), *Gammaproteobacteria* (3 strains), and *Deltaproteobacteria* (1 strain). The HTC isolates belonging to the *Alphaproteobacteria* were affiliated with 22 phylogenetic groups comprising five orders (*Caulobacterales, Rhizobiales*, *Rhodobacterales*, *Rhodospirillales*, and *Sphingomonadales*) (Figure 1A and Supplementary Table 3), all of which are known to be commonly found in aquatic ecosystems (Newton et al., 2011; Hayden and Beman, 2016; Keshri et al., 2018). Among the 62 alphaproteobacterial isolates, 44 strains were cultivated from the surface layer of Lake Soyang and 27 strains formed colonies on R2A-based media. Although the culturability of some HTC isolates appeared to be depth- and season-dependent (i.e., all 9 isolates of *Hirschia* were found exclusively in summer), these trends cannot be generalized due to the small number of isolates for each phylogenetic group. Further, although many isolates of *Alphaproteobacteria* belonged to well-defined genera (*Caulobacter*, *Brevundimonas*, *Phenylobacterium*, *Methylorosula*, *Bradyrhizobium*, *Afipia*, *Rhodobacter*, *Defluviimonas*, *Hirschia*, *Ferrovibrio*, *Novosphingobium, Sphingomonas*, and *Sandarakinorhabdus*), 22 isolates belonged to as-yet-uncultured lineages such as A0839, Alpha1, FukuN57, MNG7, Wr0007, and GOBB3-C201. In the SILVA database, the A0839 lineage currently contains > 2,300 16S rRNA gene sequences of uncultured bacteria retrieved from diverse aquatic or terrestrial habitats including an Antarctic lake (Nakai et al., 2012) and a metal-rich freshwater reservoir (Stein et al., 2002). Given that strain IMCC27047 of the A0839 lineage formed colonies, additional studies should be conducted to characterize the taxonomy and ecological roles of this uncultivated bacterial lineage. In contrast, isolates of the Alpha1, FukuN57, MNG7, and Wr0007 lineages did not produce colonies on R2A agar but resuscitated well from frozen glycerol stocks, which can be further used for liquid culture-based ecophysiological studies. In this HTC trial, members of the LD12 lineage, the predominant freshwater alphaproteobacterial clade, could not be isolated despite being highly abundant in Lake Soyang waters. Only a single isolate of the LD12 clade, LSUCC0530 (“*Candidatus* Fonsibacter”), has been reportedly isolated from brackish water (∼5‰ salinity) (Henson et al., 2018), suggesting that cultivating LD12 strains from freshwater lakes will likely require highly optimized culture media and growth conditions.

Only three strains of *Gammaproteobacteria* and one strain of *Deltaproteobacteria* were isolated throughout the study (Figure 1A). The three *Gammaproteobacteria* strains belonged to the genera *Acinetobacter, Enhydrobacter*, and *Arenimonas* and were isolated in autumn or winter. Strain IMCC30040, the sole isolate belonging to *Deltaproteobacteria* identified herein was assigned to the genus *Sorangium*, which is a soil-borne bacterial genus rarely found in aquatic systems.

### Betaproteobacteria

Within the phylum *Proteobacteria*, the class Betaproteobacteria is known to dominate various freshwater lakes worldwide (Lindstrom et al., 2005; Newton et al., 2011; Jezbera et al., 2012; Okazaki et al., 2021). In our HTC experiments, 449 strains (74.2% of the total isolates) belonged to 26 phylogenetic groups of *Betaproteobacteria*, thus also demonstrating that *Betaproteobacteria* was the most abundant and diverse group in the cultured representatives (Figure 1B and Supplementary Table 3). Most betaproteobacterial isolates were assigned to the orders *Burkholderiales*, *Methylophilales*, and *Nitrosomonadales*. The phylogenetic groups that encompassed 10 or more HTC isolates included LimB (80 isolates), LimD (36), a new *Limnohabitans* group (Lim 2) represented by IMCC26036 (52) (Figure 1B), *Rhodoferax_Albidiferax* (53), PnecA (20), PnecB2 (24), PnecC (39), and a new *Polynucleobacter* group (Pnec1) represented by IMCC26046 (13) (Supplementary Figure 4) of the order *Burkholderiales*, as well as the LD28 freshwater group (84) of the order *Methylophilales*. These groups were all found to belong to four well-known freshwater bacterial genera: *Limnohabitans, Polynucleobacter, Rhodoferax* (including *Albidiferax*), and “*Candidatus* Methylopumilus” (LD28 freshwater group). In addition to these groups, some HTC isolates were identified as members of the MWH-UniP1, GKS98, and TRA3-20 groups that currently do not have any cultured representatives. At least one strain from the three groups formed colonies, which will likely prove to be an important resource for future taxonomic and genomic studies. In total, 68 betaproteobacterial isolates exhibited colony-forming capacities, and most of them (50 isolates) were identified to be members of the genus *Polynucleobacter* (Supplementary Table 3).

The majority (174 isolates, 38.7%) of *Betaproteobacteria* belonged to groups related to the genus *Limnohabitans* (Figures 1B, 2). The genus *Limnohabitans* is known to inhabit diverse freshwater ecosystems (Jezbera et al., 2013; Shabarova et al., 2017) and is currently composed of four published species (Hahn et al., 2010a, b; Kasalický et al., 2010). Based on the phylogeny of the 35 isolates of the genus *Limnohabitans*, the *Limnohabitans* classification system consisting of five LimA-E lineages was proposed (Kasalický et al., 2013). Upon applying this classification scheme to the HTC isolates obtained in this study, 174 *Limnohabitans* isolates were found to be affiliated with the LimB, LimC, and LimD lineages, as well as the two newly identified Lim1 and Lim2 lineages herein (Figure 2). Concretely, these two novel *Limnohabitans* lineages were named “Lim1” and “Lim2” because the HTC isolates that were classified as such were not associated with the five previously defined lineages and instead formed separate lineages. The Lim1 lineage contained four HTC isolates and the Lim2 lineage consisted of 52 HTC isolates. In contrast with the colony-producing *L. curvus* and *L. australis* of lineage LimA and *L. parvus* and *L. planktonicus* of lineage LimC, all HTC isolates of the *Limnohabitans* group except for three isolates of the Lim2 lineage could not grow on R2A-based media. Given that the genus *Limnohabitans* is generally recognized as an ecologically important but not easily cultivable bacterioplankton group (Glockner et al., 2000), the inability of many of the *Limnohabitans* isolates characterized herein to form colonies was consistent with previous findings. Furthermore, given that the LimD lineage does not have any cultured isolates and that this study is the first to describe the Lim 1–2 lineages, the 174 novel strains isolated herein may provide an important basis for future freshwater microbial characterizations, such as the recently identified aerobic anoxygenic photosynthetic potential of the genus *Limnohabitans* (Kasalický et al., 2018).

The genus *Polynucleobacter* was the second largest group within the HTC isolates (96 isolates) and was further distributed into the lineages PnecA (20 isolates), PnecB2 (24), PnecC (39), and a newly identified lineage named Pnec1 (13) (Figure 1B and Supplementary Figure 4). Pnec1 was considered a new lineage because 13 HTC isolates sharing 100% of their 16S rRNA gene sequences could not be assigned to any of the four known PnecA-D clades (Wu and Hahn, 2006a; Newton et al., 2011). The genus *Polynucleobacter* is a ubiquitous and abundant freshwater betaproteobacterial group (Hahn et al., 2015, 2016a), which currently comprises 17 validly published species with *P. necessarius* as the type species (Hahn et al., 2016b). Eleven narrowly diversified species belong to the PnecC lineage, whereas the PnecA, PnecB, and PnecD lineages contain only one, three, and two validly published species, respectively. Most of the HTC isolates (36/39) assigned to PnecC and Pnec1 (10/13) produced colonies. In contrast, only a few isolates of PnecA (4/20) formed colonies on R2A media and PnecB2 isolates did not form colonies, suggesting that each lineage has highly unique growth conditions. Some members of the genus *Polynucleobacter* are known to exhibit highly predictable seasonal dynamics depending primarily on water temperature (Wu and Hahn, 2006b). Likewise, although the HTC strains of *Polynucleobacter* were isolated from all seasons and depths, each Pnec lineage displayed a different cultivation pattern. For example, 20 PnecA strains were isolated in spring and summer, whereas 24 PnecB2 strains were isolated in autumn and winter. Interestingly, although the 16S rRNA gene sequences of 13 isolates belonging to the Pnec1 clade were identical, they were retrieved in all seasons mostly from a 50 m depth.

In this study, a total of 88 strains were assigned to the family *Methylophilaceae*. This family is one of the key methylotrophic bacterial groups in freshwater and marine habitats (Chistoserdova, 2015). The family *Methylophilaceae* currently comprises four genera (*Methylotenera, Methylobacillus, Methylophilus*, and *Methylovorus*), in addition to the LD28 freshwater group (“*Candidatus* Methylopumilus planktonicus”) (Newton et al., 2011; Salcher et al., 2015), OM43 marine group (Giovannoni et al., 2008), and PRD01a001B group (“*Candidatus* Methylopumilus planktonicus) (Salcher et al., 2015), the members of which might have transitioned from freshwater sediments to the pelagic environment (Salcher et al., 2019). These HTC isolates belonged to the LD28 lineage (84 isolates), PRD01a001B lineage (1), and the genus *Methylotenera* (3) (Figure 1B and Supplementary Figure 5). Interestingly, all strains assigned to the family *Methylophilaceae* were isolated in winter, which may be due to their psychrophilic physiology, as observed in Lake Zurich (Salcher et al., 2015). The 16S rRNA gene sequence diversity of 84 strains of the LD28 lineage was relatively low (98.5-100%) and many sequences shared 100% similarity, resulting in only 16 unique sequences out of a total of 84 (Supplementary Figure 5). Among them, strain IMCC30216 shared 100% 16S rRNA gene sequence similarity with “*Candidatus* Methylopumilus planktonicus,” whereas other HTC isolates displayed different phylogenetic relationships with other European lake isolates. Future genome-based taxonomic studies may reveal whether the global distribution of LD28 bacteria displays signs of endemism. In total, four HTC isolates of the LD28, PRD01a011B, and *Methylotenera* lineages formed colonies on 1/10R2A media, which will facilitate the polyphasic taxonomy of these isolates. Given that one of the LD28 isolates cultured in our study grew well in a methanol-supplemented artificial freshwater medium and was infected by a bacteriophage strain (Moon et al., 2017), numerous LD28 strains could be used as bacterial hosts for the isolation of lytic phages specific to this lineage.

### Actinobacteria

The phylum *Actinobacteria* is considered the most abundant bacterial group in freshwater lakes, often accounting for > 50% of limnetic bacterioplankton (Glockner et al., 2000; Zwart et al., 2002; Warnecke et al., 2004). In our HTC experiment, 49 HTC isolates were affiliated with 11 phylogenetic groups within three actinobacterial orders including *Frankiales* (5 strains), *Acidimicrobiales* (19 strains), and *Gaiellales* (25 strains) (Figures 1C, 3 and Supplementary Table 3). However, none of the 49 actinobacterial strains obtained in this study formed colonies on R2A-based agar plates.

All five strains affiliated with the order *Frankiales* belonged to the acI lineage, the predominant freshwater actinobacterial group (Zwart et al., 2002; Warnecke et al., 2004). The HTC isolates of the acI lineage were identified as members of the acI-A1 (IMCC30251, IMCC30273), acI-A4 (IMCC26103), acI-B1 (IMCC26068), and acI-C1 (IMCC26077) tribes (Figure 3). When the five acI strains were first isolated, all initial cultures failed to resuscitate from frozen glycerol stocks. Among these isolates, however, complete genome sequences were later obtained from strains IMCC26103 and IMCC26077 (Kang et al., 2017), after which 16 genomes of the acI clade were also retrieved from Lake Zurich (Neuenschwander et al., 2018). Recently, our research group successfully cultivated strain IMCC26103 in a freshwater-based medium supplemented with catalase and designated this strain as “*Candidatus* Planktophila aquatilis,” as well as strain IMCC25003, which we named “*Candidatus* Planktophila rubra” (Kim et al., 2019). We also recently isolated 75 acI strains assigned to 8 acI tribes (acI-A1, A2, A4, A5, A6, A7, B1, B4, C1, and C2) and confirmed that at least each representative strain of each tribe could resuscitate from glycerol stocks (Kim et al., 2020). Therefore, our results established a precedence for the use of HTC trials for freshwater bacterial isolation and provided insights into the mechanisms that led to the “unculturability” of some microorganisms, all of which will surely promote the continued isolation of uncultured bacteria.

Among the 19 strains assigned to *Acidimicrobiales*, 17 strains belonged to the acIV lineage (Figure 1C), which is another typical and abundant freshwater bacterial group that was first discovered in samples obtained from Adirondack Mountain Lake (Hiorns et al., 1997), named by Warnecke et al. (2004), and subdivided into four subclades (acIV-A to D) (Humbert et al., 2009; Philosof et al., 2009; Newton et al., 2011; Martinez-Garcia et al., 2012b; Okazaki et al., 2021). The families *Ilumatobacteriaceae* (Matsumoto et al., 2009) and *Iamiaceae* (Kurahashi et al., 2009; Liu et al., 2021) and the genus “*Candidatus* Limnosphaera” (Kim et al., 2017) are considered the nearest taxa to the acIV lineage. Moreover, although the acIV lineage has been widely identified in many freshwater lakes (Humbert et al., 2009; Philosof et al., 2009; Martinez-Garcia et al., 2012b; Okazaki et al., 2021), no cultured representatives have been reported to date. In our HTC trials, 17 strains of the acIV lineage were identified as members of the acIV-A2 (6 strains), acIV-B (IMCC30049), acIV-C (IMCC26084), and acIV-D (9 strains) tribes (Figure 3). Furthermore, although acIV-D strains were isolated in different seasons and depths, different members of the acIV-A2, -B, and -C tribes were cultivated from each specific season and depth. Considering the topology of the phylogenetic trees (Figures 1C,3), the acIV lineage, which was previously proposed as a monophyletic group comprising four sub-lineages, should be divided into an acIV-ABC clade (acIV *sensu stricto*) and an acIV-D clade, which will be eventually renamed (Newton et al., 2011). Two actinobacterial strains were identified as “*Candidatus* Limnosphaera” (i.e., a sister group of acIV-D) (Kim et al., 2017) based on the characteristics of strain IMCC26207 isolated in this study. Although the HTC strains classified as acIV-B and -C were not revived from glycerol stocks, the genetic information currently compiled from these bacteria may provide important insights into their potential culture strategies, as seen in the successful cultivation of acI bacteria.

In the order *Gaiellales*, a total of 25 HTC isolates were affiliated with two new groups defined in this study for which cultured representatives have not yet been reported. These two groups were named “unidentified *Gaiellales* 1” (3 isolates) represented by strain IMCC29153 and “unidentified *Gaiellales* 2” (22 isolates) represented by strain IMCC29033 (Figure 3). In the phylogenetic analysis, three HTC strains of the unidentified *Gaiellales* 1 group were clustered with the genus *Gaiella* (Albuquerque et al., 2011), the type and only genus of the order *Gaiellales*, whereas 22 HTC strains of the unidentified *Gaiellales* 2 group formed an independent clade separate from the genus *Gaiella.* The unidentified *Gaiellales* 2 group included many previously uncultured members of the AKIW543 clade, comprising sequences obtained from freshwater, marine, and soil environments.

### *Bacteroidetes* and *Verrucomicrobia*

The number of HTC isolates assigned to the family *Flavobacteriaceae* was the highest among the phylum *Bacteroidetes* (27 of 36 strains of *Bacteroidetes*) (Figure 1). Moreover, all isolated *Flavobacteriaceae* strains in this study were assigned to the genus *Flavobacterium*, comprising over 330 species from diverse habitats.^1^ Although the members of the genus *Flavobacterium* are known to form colonies, 12 *Flavobacterium* strains could not produce colonies. Another nine HTC strains were identified as members of *Pseudarcicella, Ferruginibacter*, LiUU-11-161, env. OPS 17, and uncultured *Cytophagaceae*. Among the 36 *Bacteroidetes* strains, 34 strains were isolated from surface water, suggesting that these bacteria might depend on phytoplankton as a source of high molecular-weight organic matter (Williams et al., 2013; Bennke et al., 2016).

In total, five HTC isolates were assigned to three groups of the phylum *Verrucomicrobia*: OPB35_soil group (*Pedosphaeraceae*; 3 strains), *Opitutae* (1 strain), and *Prosthecobacter* (1 strain) (Figure 1C and Supplementary Figure 6). However, neither of these isolates formed colonies and only one strain (IMCC26040) could be revived from glycerol stocks, which poses important challenges for the cultivation of *Verrucomicrobia* isolates. The OPB35_soil group is known to degrade complex polymeric substances such as laminarin or xylan (Martinez-Garcia et al., 2012a; Cardman et al., 2014). Therefore, the cultivated strain IMCC26040 may enable the study of the ecological roles of the OPB35_soil group in the degradation of naturally occurring polymers.

### Bacterial Revival From Frozen Glycerol Stocks

**FIGURE 4:**
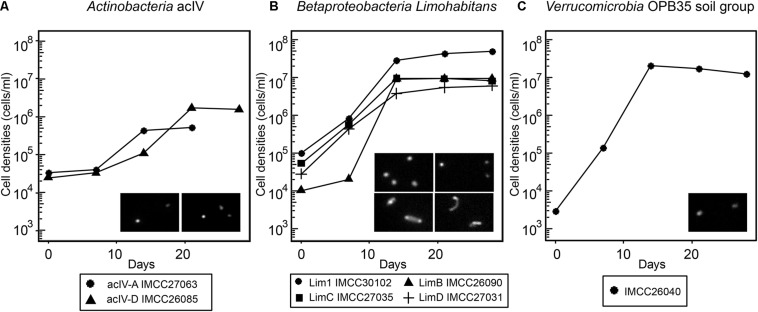
Growth curves of selected HTC isolates revived from frozen glycerol stocks. **(A)** acIV-A and acIV-D. **(B)** Lim1, LimB, LimC, and LimD. **(C)** OPB35 soil group. The HTC isolates are prefixed with the “IMCC” abbreviation (Inha Microbe Culture Collection). The inset images are DAPI-stained micrographs taken with an epifluorescence microscope; **(A)** left, IMCC27063; right, UMCC26085. **(B)** upper left, IMCC30102; upper right, IMCC26090; below left, IMCC27035; below right, IMCC27031.

As exemplified by the cultivation of the acI bacteria (Kim et al., 2019), some initial HTC cultures may not revive from frozen glycerol stocks, thus hampering further genomic and physiological analyses. Therefore, to design physiological and multi-omics-based experiments for further characterization of freshwater bacterioplankton, it is important to determine whether the HTC isolates initially grown in natural freshwater-based media can form colonies or re-grow after being thawed from frozen stocks stored at −80°C. Of the 71 phylogenetic groups encompassing the HTC strains isolated herein, a total of 119 strains representing 25 groups produced colonies. The 25 phylogenetic groups that had colony-former(s) were excluded from the reviving experiment, assuming that the colonies formed in agar plates could be restocked as glycerol suspension and these stocks are known to regrow well after thawing. As a result, representative strains from each 46 phylogenetic group were selected and used for the revival experiments, of which 29 strains representing 22 groups revived successfully from glycerol stocks (Supplementary Figure 7). The phylogenetic groups to which the revived non-colony-forming strains belonged included several ecologically relevant freshwater bacterial groups such as FukuN57, MNG7, and Wr0007 (*Alphaproteobacteria*); PnecB2, Lim1, and LimB (*Betaproteobacteria*); acIV, uncultured *Gaiellales* group 2, and “*Candidatus* Limnosphaera” (*Actinobacteria*); and OPB35_soil group (*Verrucomicrobia*). Among these, two strains of acIV, four strains of *Limnohabitans*, and strain IMCC26040 of the OPB35_soil group were selected for growth curve determination. The seven strains reached their maximum cell densities at 10-20 days of incubation in axenic conditions, exhibiting fastidious but stable growth characteristics (Figure 4). Furthermore, some of the bacterial strains regrown in the reviving experiments had been reported in previous taxonomic and genomic studies. Strain IMCC26207 was reported as a new taxon of *Actinobacteria* named “*Candidatus* Limnosphaera” (Kim et al., 2017). Strain IMCC26223 of the genus *Flavobacterium* and strain IMCC26026 of the phylum *Bacteroidetes* have also been reported as new species in previous polyphasic taxonomy studies (Nam et al., 2017; Park et al., 2017). Additionally, strains IMCC26059 and IMCC26218 of the class *Betaproteobacteria* were used to isolate specific lytic bacteriophages (Moon et al., 2015, 2018). Therefore, as exemplified by the aforementioned studies, the HTC isolates obtained herein will surely contribute to our understanding of freshwater microbial ecology.

### Comparison Between the Cultured HTC Isolates and the Uncultured Bacterial Community

**FIGURE 5:**
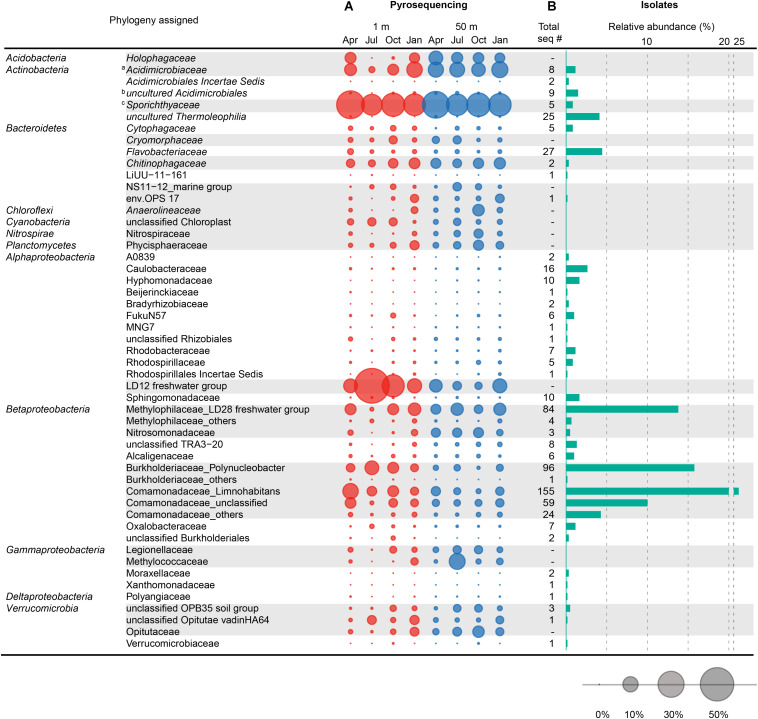
Comparison between the family-level taxonomic composition of the bacterial community structure obtained by pyrosequencing and the composition of the bacterial isolates cultured by HTC. **(A)** Seasonal variation of the bacterial community structure at the family level observed at 1 and 50 m depths in Lake Soyang, as characterized by 16S rRNA gene pyrosequencing. **(B)** Phylogenetic composition and abundance of 605 axenic HTC isolates at the family level. The bubbles represent the percentage of pyrotags of each taxonomic group in each sample. Taxonomic groups with gray shades represent major phylogenetic groups that occupied > 1% of the total bacterial community in eight samples or > 2.5% of the bacterial community in each sample. Taxonomic classification of 16S rRNA gene sequences of HTC isolates and pyrotags was determined using the Mothur software based on the SILVA taxonomy database. ^*a*^ Contains the acIV-A, -B, and -C lineages. ^*b*^ Contains acIV-D lineage. ^*c*^ Contains the acI lineage.

**FIGURE 6:**
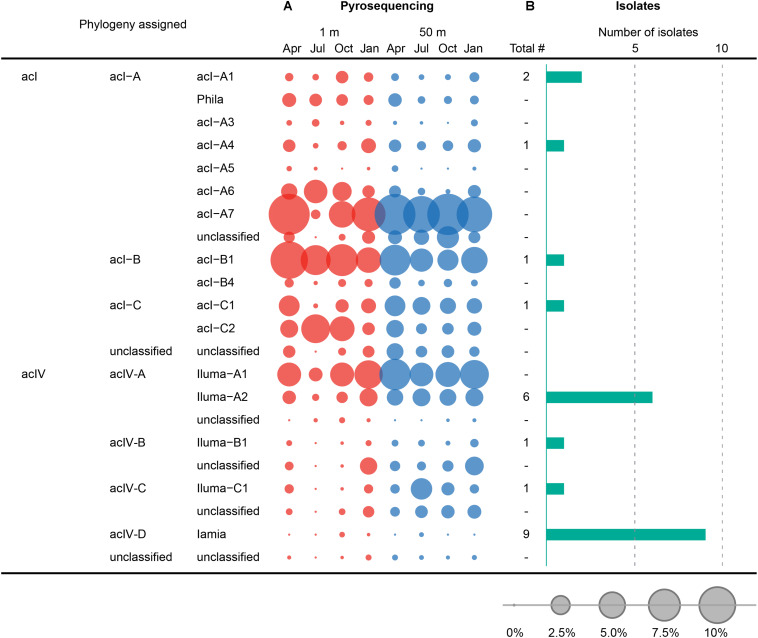
Comparison between the composition of HTC isolates and pyrotags at the tribe level belonging to the acI and acIV lineages of the phylum *Actinobacteria*. **(A)** Seasonal variation of tribes of the acI and acIV lineages, observed at 1 and 50 m depths in Lake Soyang, as characterized by 16S rRNA gene pyrosequencing. **(B)** Number of HTC isolates belonging to tribes of the acI and acIV linages. The bubbles represent the percentage of pyrotags of each taxonomic group in each sample. Taxonomic classification of 16S rRNA gene sequences of HTC isolates and pyrotags was determined using the freshwater-specific FreshTrain database (Rohwer et al., 2018).

A total of 63,954 high-quality 16S rRNA gene sequences comprising 14,231 unique sequences and 1,360 (2% cut-off) OTUs were obtained from eight freshwater samples used as inocula during the HTC trials (Supplementary Figure 3). The freshwater bacterial community structure elucidated by this culture-independent analysis was compared with the composition of the HTC isolates, then visualized at the family (Figure 5) and tribe levels for *Actinobacteria* (Figure 6).

Among the 29 phyla identified, eight phyla (*Proteobacteria*, *Actinobacteria*, *Bacteroidetes*, *Verrucomicrobia*, *Acidobacteria*, *Planctomycetes*, *Chloroflexi*, and *Cyanobacteria*) accounted for more than 1.0% of the total reads. The phyla *Proteobacteria* and *Actinobacteria* were always dominant in all samples, accounting for 35.4−67.1 and 20.3−40.8% in 1 m depth samples, and 26.7−37.8 and 29.8−42.0% in 50 m depth samples, followed by *Bacteroidetes* (average 8.5%), *Verrucomicrobia* (5.5%), *Acidobacteria* (4.1%), *Planctomycetes* (2.7%), *Chloroflexi* (2.1%), and *Cyanobacteria* (1.9%) (Supplementary Figure 8). Within the proteobacterial community, *Alphaproteobacteria* (average 17.8% of the total sequences) and *Betaproteobacteria* (17.5%) were the predominant classes in all samples, whereas *Gammaproteobacteria* (3.7%) and *Deltaproteobacteria* (1.0%) were only present in far lower proportions. However, their abundance varied with season and water depth.

Bacterial community structure analysis based on hierarchical clustering with UPGMA and PCoA using Bray-Curtis distance dissimilarity indicated that the bacterial communities of the surface layer (1 m) of Lake Soyang exhibited more dramatic seasonal variations than those of the hypolimnetic layer (50 m) (Supplementary Figure 9). Therefore, while the bacterial communities in winter and spring clustered together based on season, those in summer and autumn clustered primarily based on sampling depths. Particularly, the bacterial community of the summer surface sample was very distinct from the communities of the remaining samples, as easily predicted from the high proportion of *Alphaproteobacteria* in the summer surface (Supplementary Figure 8). These variations in bacterial community structure are thought to be largely attributable to the monomictic characteristics of the lake (i.e., the formation of a strong thermocline in summer and complete mixing during the winter season). In fact, the community structures of the epilimnion and hypolimnion zones were very similar in the winter season.

The comparison between the uncultured bacterial community of Lake Soyang and the isolates cultured via HTC indicated that only small fractions of the freshwater bacterial community could be enriched and grown at the specific HTC conditions (Figure 5). Out of the eight phyla for which sequencing reads exceeded 1.0% of the total pyrosequencing reads, only the members of four phyla (*Proteobacteria*, *Actinobacteria*, *Bacteroidetes*, and *Verrucomicrobia*) could be cultivated. Furthermore, members of the class *Betaproteobacteria* were abundantly found in both the culture-independent analyses (17.5% of the total bacterial community) and the HTC experiment (74.2% of total HTC isolates), suggesting that the HTC culture conditions favored the growth of *Betaproteobacteria*. Similarly, the *Limnohabitans*, *Polynucleobacter*, and “*Candidatus* Methylopumilus” genera, which accounted for high proportions of the HTC isolates, were also abundantly present in the bacterial community of Lake Soyang (Figure 5). Additionally, supplementation of growth media with bacteria-accessible carbon sources (e.g., methanol) may facilitate the cultivation of members of the *Betaproteobacteria* class, as observed in the methylotrophic LD28 lineage. In contrast, phylogenetic analyses based on family-level taxa indicated that members of some families within the major four phyla could not be isolated despite their dominance in the bacterial community. For example, although the LD12 freshwater *Alphaproteobacteria* group accounted for 53.9% of the bacterial community of the surface waters in summer, the cultivation of LD12 bacteria failed in the HTC conditions. This discrepancy between the uncultured bacterial dominance and the scarcity of cultured isolates was also observed in the actinobacterial acI and acIV lineages (Figure 6). The family *Sporichthyaceae* (*Actinobacteria*), which was primarily represented by the acI lineage, appeared to be consistently abundant regardless of season or depth (Figure 5). In contrast, the diversity of the uncultured acI tribes varied significantly depending on season and depth (Figure 6). The abundance of the uncultured members of acI-A7 decreased dramatically in the surface waters in summer, whereas that of acI-C2 increased in the same season and depth. Further, although we isolated five strains of the acI lineage, neither of the members of these abundant tribes could be isolated, including acI-A7 and acI-C2. Similarly, none of the acIV-A lineage members could be cultivated despite their abundance in the uncultured bacterial community, whereas nine strains of the acIV-D lineage were isolated from relatively small fractions of the uncultured community.

To determine the proportion of the total bacterial community represented by the HTC isolates, the 605 sequences of the HTC isolates were queried via BLASTn analysis against eight pyrosequencing data sets retrieved from Lake Soyang. These analyses indicated that 26.7 and 24.5% of the pyrosequencing reads corresponded to the 16S rRNA gene sequences of the HTC isolates based on 97.0 and 98.7% sequence similarity thresholds, respectively (Supplementary Table 4). The phylogenetic groups corresponding to the sequences of the HTC isolates included many *Actinobacteria* and *Betaproteobacteria* groups such as acI-B1 (5.8%), the LD28 freshwater group (4.6%), and LimB (2.5%), confirming that these groups were successfully cultivated using the HTC method employed herein.

Overall, our results indicated that the bacterial community composition of Lake Soyang was similar to that of many lacustrine environments worldwide (Newton et al., 2011; Jezbera et al., 2012; Okazaki et al., 2021). Particularly, many of the dominant phylogenetic groups in Lake Soyang were consistent with those of similar aquatic environments, including the acI, acIV, LD12, LD28, *Polynucleobacter*, and *Limnohabitans* lineages, whose pyrosequencing tags were largely dominant. The HTC method using media based on ambient lake water allowed for the cultivation of these dominant bacterioplankton groups. However, some of the major lineages and tribes including LD12, acI-A6, -A7, -B4, and -C2 could not be cultivated with this approach. Considering the small number of HTC isolates belonging to the major bacterial lineages observed in this study, the medium composition and culture conditions used herein may have not favored some of the aforementioned lineages. Therefore, to obtain stably growing axenic cultures of fastidious microorganisms, future studies must focus on the development of cultivation strategies based on genomic and ecological data. One remarkable example of such an approach is the recent successful cultivation of the genome-streamlined acI lineage by supplementing its growth media with chemical compounds required for their growth, including reduced sulfurs, amino acids, vitamins, and catalase (Kang et al., 2017; Kim et al., 2019, 2020). This successful cultivation of the acI lineage would not be possible if the HTC campaigns reported in this study were not performed previously, thus highlighting the power and efficacy of microbial ecology research based on both culture-dependent and -independent approaches.

## Conclusion

In this study, we conducted HTC experiments and tag-encoded pyrosequencing analyses using water samples collected from the epilimnion and hypolimnion of Lake Soyang over four seasons. This resulted in the isolation of more than 600 bacterial strains and enabled the characterization of the bacterial community structure of Lake Soyang, a freshwater reservoir. The Lake Soyang bacterial community was comprised of phylogenetically diverse bacterioplankton groups, most of which belonged to cosmopolitan freshwater bacterioplankton lineages, but whose populations varied dramatically in a season- and depth-dependent fashion. The isolates cultured via HTC were assigned to many predominant and previously uncultivated or underrepresented phylogenetic groups, including multiple tribes of the acI, acIV, LD28, FukuN57, MNG9, and TRA3-20 lineages. Nevertheless, despite the successful cultivation of previously uncultivated freshwater bacterioplankton groups, many abundant bacterial lineages such as LD12 and *Acidobacteria* could not be cultured using HTC. Therefore, additional studies are required to optimize the HTC growth conditions of these freshwater lineages by integrating the ever-increasing wealth of related genomic and ecological data.

## Data Availability Statement

The 16S rRNA gene sequences of the HTC isolates were deposited in the GenBank/EMBL/DDBJ databases under accession numbers MW884569–MW885173. The accession numbers for each sample are also listed in Supplementary Data. The pyrosequencing data reported herein were also submitted to the Sequence Read Archive (SRA) database under accession number SRR14278392–SRR1427839.

## Author Contributions

SK and MI performed the wet experiments. SK and IK performed bioinformatic data analyses. IK and J-CC designed and directed the study. SK, MI, IK, and J-CC analyzed the data and wrote the manuscript. All authors contributed to the article and approved the submitted version.

## Conflict of Interest

The authors declare that the research was conducted in the absence of any commercial or financial relationships that could be construed as a potential conflict of interest.

## Publisher’s Note

All claims expressed in this article are solely those of the authors and do not necessarily represent those of their affiliated organizations, or those of the publisher, the editors and the reviewers. Any product that may be evaluated in this article, or claim that may be made by its manufacturer, is not guaranteed or endorsed by the publisher.

## Footnotes

1
http://lpsn.dsmz.de

## References

[B1] Albuquerque L. Franca L. Rainey F. A. Schumann P. Nobre M. F. da Costa M. S. (2011). *Gaiella occulta* gen. nov., sp. nov., a novel representative of a deep branching phylogenetic lineage within the class Actinobacteria and proposal of Gaiellaceae fam. nov. and Gaiellales ord. nov. *Syst. Appl. Microbiol.* 34 595–599. 10.1016/j.syapm.2011.07.001 21899973

[B2] Altschul S. F. Madden T. L. Schaffer A. A. Zhang J. H. Zhang Z. Miller W. (1997). Gapped BLAST and PSI-BLAST: a new generation of protein database search programs. *Nucleic Acids Res.* 25 3389–3402. 10.1093/nar/25.17.3389 9254694 PMC146917

[B3] APHA (2005). *Standard Methods for the Examination of Water and Waste Water*, 21st Edn. Washington, DC: American Public Health Association.

[B4] Bennke C. M. Kruger K. Kappelmann L. Huang S. Gobet A. Schuler M. (2016). Polysaccharide utilisation loci of Bacteroidetes from two contrasting open ocean sites in the North Atlantic. *Environ. Microbiol.* 18 4456–4470. 10.1111/1462-2920.13429 27348854

[B5] Boscaro V. Felletti M. Vannini C. Ackerman M. S. Chain P. S. G. Malfatti S. (2013). Polynucleobacter necessarius, a model for genome reduction in both free-living and symbiotic bacteria. *Proc. Nat. Acad. Sci. U.S.A.* 110 18590–18595. 10.1073/pnas.1316687110 24167248 PMC3831957

[B6] Button D. K. Schut F. Quang P. Martin R. Robertson B. (1993). Viability and isolation of marine bacteria by dilution culture: theory, procedures, and initial results. *Appl. Environ. Microbiol.* 59 881–891. 10.1128/AEM.59.3.881-891.1993 16348896 PMC202203

[B7] Cardman Z. Arnosti C. Durbin A. Ziervogel K. Cox C. Steen A. D. (2014). Verrucomicrobia are candidates for polysaccharide-degrading bacterioplankton in an Arctic Fjord of Svalbard. *Appl. Environ. Microbiol.* 80 3749–3756. 10.1128/aem.00899-14 24727271 PMC4054139

[B8] Chistoserdova L. (2015). Methylotrophs in natural habitats: current insights through metagenomics. *Appl. Microbiol. Biotechnol.* 99 5763–5779. 10.1007/s00253-015-6713-z 26051673

[B9] Cho J. C. Giovannoni S. J. (2004). Cultivation and growth characteristics of a diverse group of oligotrophic marine Gammaproteobacteria. *Appl. Environ. Microbiol.* 70 432–440. 10.1128/Aem.70.1.432-440.2004 14711672 PMC321273

[B10] Cho J. C. Stapels M. D. Morris R. M. Vergin K. L. Schwalbach M. S. Givan S. A. (2007). Polyphyletic photosynthetic reaction centre genes in oligotrophic marine Gammaproteobacteria. *Environ. Microbiol.* 9 1456–1463. 10.1111/j.1462-2920.2007.01264.x 17504483

[B11] Connon S. A. Giovannoni S. J. (2002). High-throughput methods for culturing microorganisms in very-low-nutrient media yield diverse new marine isolates. *Appl. Environ. Microbiol.* 68 3878–3885. 10.1128/Aem.68.8.3878-3885.2002 12147485 PMC124033

[B12] Dixon P. (2003). VEGAN, a package of R functions for community ecology. *J. Veg. Sci.* 14 927–930. 10.1111/j.1654-1103.2003.tb02228.x

[B13] Giovannoni S. J. Hayakawa D. H. Tripp H. J. Stingl U. Givan S. A. Cho J. C. (2008). The small genome of an abundant coastal ocean methylotroph. *Environ. Microbiol.* 10 1771–1782. 10.1111/j.1462-2920.2008.01598.x 18393994

[B14] Glockner F. O. Zaichikov E. Belkova N. Denissova L. Pernthaler J. Pernthaler A. (2000). Comparative 16S rRNA analysis of lake bacterioplankton reveals globally distributed phylogenetic clusters including an abundant group of actinobacteria. *Appl. Environ. Microbiol.* 66 5053–5065. 10.1128/aem.66.11.5053-5065.2000 11055963 PMC92419

[B15] Häder D.-P. Williamson C. E. Wängberg S. -Å Rautio M. Rose K. C. Gao K. (2015). Effects of UV radiation on aquatic ecosystems and interactions with other environmental factors. *Photochem. Photobiol. Sci.* 14 108–126. 10.1039/c4pp90035a 25388554

[B16] Hahn M. W. (2003). Isolation of strains belonging to the cosmopolitan *Polynucleobacter necessarius* cluster from freshwater habitats located in three climatic zones. *Appl. Environ. Microbiol.* 69 5248–5254. 10.1128/Aem.69.9.5248-5254.2003 12957910 PMC194981

[B17] Hahn M. W. Jezberova J. Koll U. Saueressig-Beck T. Schmidt J. (2016a). Complete ecological isolation and cryptic diversity in *Polynucleobacter* bacteria not resolved by 16S rRNA gene sequences. *ISME J.* 10 1642–1655. 10.1038/ismej.2015.237 26943621 PMC4913878

[B18] Hahn M. W. Kasalicky V. Jezbera J. Brandt U. Jezberova J. Simek K. (2010a). *Limnohabitans curvus* gen. nov., sp. nov., a planktonic bacterium isolated from a freshwater lake. *Int. J. Syst. Evol. Microbiol.* 60, 1358–1365. 10.1099/ijs.0.013292-0 19671731 PMC3091418

[B19] Hahn M. W. Kasalicky V. Jezbera J. Brandt U. Simek K. (2010b). *Limnohabitans australis* sp. nov., isolated from a freshwater pond, and emended description of the genus *Limnohabitans*. *Int. J. Syst. Evol. Microbiol.* 60, 2946–2950. 10.1099/ijs.0.022384-0 20118294 PMC3031073

[B20] Hahn M. W. Koll U. Jezberova J. Camacho A. (2015). Global phylogeography of pelagic *Polynucleobacter* bacteria: restricted geographic distribution of subgroups, isolation by distance and influence of climate. *Environ. Microbiol.* 17 829–840. 10.1111/1462-2920.12532 24920455 PMC4361717

[B21] Hahn M. W. Schmidt J. Pitt A. Taipale S. J. Lang E. (2016b). Reclassification of four *Polynucleobacter necessarius* strains as representatives of *Polynucleobacter asymbioticus* comb. nov., *Polynucleobacter duraquae* sp. nov., *Polynucleobacter yangtzensis* sp. nov. and *Polynucleobacter sinensis* sp. nov., and emended description of *Polynucleobacter necessarius*. *Int. J. Syst. Evol. Microbiol.* 66 2883–2892. 10.1099/ijsem.0.001073 27064460 PMC5018217

[B22] Hall T. A. (1999). BioEdit: a user-friendly biological sequence alignment editor and analysis program for Windows 95/98/NT. *Nucleic Acids Symp. Ser.* 41 95–98.

[B23] Hayden C. J. Beman J. M. (2016). Microbial diversity and community structure along a lake elevation gradient in Yosemite National Park, California, USA. *Environ. Microbiol.* 18 1782–1791. 10.1111/1462-2920.12938 26058326

[B24] Henson M. W. Lanclos V. C. Faircloth B. C. Thrash J. C. (2018). Cultivation and genomics of the first freshwater SAR11 (LD12) isolate. *ISME J.* 12 1846–1860. 10.1038/s41396-018-0092-2 29599519 PMC6018831

[B25] Henson M. W. Pitre D. M. Weckhorst J. L. Lanclos V. C. Webber A. T. Thrash J. C. (2016). Artificial seawater media facilitate cultivating members of the microbial majority from the Gulf of Mexico. *mSphere* 1:e00028-16. 10.1128/mSphere.00028-16 27303734 PMC4894692

[B26] Hiorns W. D. Methé B. A. Nierzwicki-Bauer S. A. Zehr J. P. (1997). Bacterial diversity in Adirondack mountain lakes as revealed by 16S rRNA gene sequences. *Appl. Environ. Microbiol.* 63 2957–2960. 10.1128/AEM.63.7.2957-2960.1997 9212443 PMC168592

[B27] Humbert J. F. Dorigo U. Cecchi P. Le Berre B. Debroas D. Bouvy M. (2009). Comparison of the structure and composition of bacterial communities from temperate and tropical freshwater ecosystems. *Environ. Microbiol.* 11 2339–2350. 10.1111/j.1462-2920.2009.01960.x 19508336

[B28] Jezbera J. Jezberová J. Kasalický V. Šimek K. Hahn M. W. (2013). Patterns of *Limnohabitans* microdiversity across a large set of freshwater habitats as revealed by reverse line blot hybridization. *PLoS One* 8:e58527. 10.1371/journal.pone.0058527 23554898 PMC3595293

[B29] Jezbera J. Jezberova J. Koll U. Hornak K. Simek K. Hahn M. W. (2012). Contrasting trends in distribution of four major planktonic betaproteobacterial groups along a pH gradient of epilimnia of 72 freshwater habitats. *FEMS Microbiol. Ecol.* 81 467–479. 10.1111/j.1574-6941.2012.01372.x 22452571 PMC4268498

[B30] Jezberova J. Jezbera J. Znachor P. Nedoma J. Kasalicky V. Simek K. (2017). The *Limnohabitans* genus harbors generalistic and opportunistic subtypes: evidence from spatiotemporal succession in a canyon-shaped reservoir. *Appl. Environ. Microbiol.* 83:e01530-17. 10.1128/AEM.01530-17 28842542 PMC5648909

[B31] Jung S. Shin M. Kim J. Eum J. Lee Y. Lee J. (2016). The effects of Asian summer monsoons on algal blooms in reservoirs. *Inland Waters* 6 406–413. 10.5268/Iw-6.3.967

[B32] Kang I. Kim S. Islam M. R. Cho J. C. (2017). The first complete genome sequences of the acI lineage, the most abundant freshwater actinobacteria, obtained by whole-genome-amplification of dilution-to-extinction cultures. *Sci. Rep.* 7:42252. 10.1038/srep42252 28186143 PMC5301498

[B33] Kasalický V. Jezbera J. Hahn M. W. Šimek K. (2013). The diversity of the *Limnohabitans* genus, an important group of freshwater bacterioplankton, by characterization of 35 isolated strains. *PLoS One* 8:e58209. 10.1371/journal.pone.0058209 23505469 PMC3591437

[B34] Kasalický V. Jezbera J. Simek K. Hahn M. W. (2010). *Limnohabitans planktonicus* sp. nov. and *Limnohabitans parvus* sp. nov., planktonic Betaproteobacteria isolated from a freshwater reservoir, and emended description of the genus *Limnohabitans*. *Int. J. Syst. Evol. Microbiol.* 60 2710–2714. 10.1099/ijs.0.018952-0 20061501 PMC3091486

[B35] Kasalický V. Zeng Y. Piwosz K. Šimek K. Kratochvilová H. Koblížek M. (2018). Aerobic anoxygenic photosynthesis is commonly present within the genus *Limnohabitans*. *Appl. Environ. Microbiology* 84:e02116-17. 10.1128/AEM.02116-17 29030444 PMC5734016

[B36] Keshri J. Pradeep Ram A. S. Nana P. A. Sime-Ngando T. (2018). Taxonomical resolution and distribution of bacterioplankton along the vertical gradient reveals pronounced spatiotemporal patterns in contrasted temperate freshwater lakes. *Microb. Ecol.* 76 372–386. 10.1007/s00248-018-1143-y 29340714

[B37] Kim B. Choi K. Kim C. Lee U. H. Kim Y. H. (2000). Effects of the summer monsoon on the distribution and loading of organic carbon in a deep reservoir, Lake Soyang, Korea. *Water Res.* 34 3495–3504. 10.1016/S0043-1354(00)00104-4

[B38] Kim O. S. Cho Y. J. Lee K. Yoon S. H. Kim M. Na H. (2012). Introducing EzTaxon-e: a prokaryotic 16S rRNA gene sequence database with phylotypes that represent uncultured species. *Int. J. Syst. Evol. Microbiol.* 62 716–721. 10.1099/ijs.0.038075-0 22140171

[B39] Kim S. Kang I. Cho J. C. (2017). Genomic analysis of a freshwater actinobacterium, “Candidatus Limnosphaera aquatica” strain IMCC26207, isolated from Lake Soyang. *J. Microbiol. Biotechnol.* 27 825–833. 10.4014/jmb.1701.01047 28173694

[B40] Kim S. Kang I. Seo J. H. Cho J. C. (2019). Culturing the ubiquitous freshwater actinobacterial acI lineage by supplying a biochemical ‘helper’ catalase. *ISME J.* 13 2252–2263. 10.1038/s41396-019-0432-x 31073214 PMC6775976

[B41] Kim S. Park M. S. Song J. Kang I. Cho J. C. (2020). High-throughput cultivation based on dilution-to-extinction with catalase supplementation and a case study of cultivating acI bacteria from Lake Soyang. *J. Microbiol.* 58 893–905. 10.1007/s12275-020-0452-2 33125668

[B42] Kim Y. Kim B. (2006). Application of a 2-dimensional water quality model (CE-QUAL-W2) to the turbidity interflow in a deep reservoir (Lake Soyang, Korea). *Lake Reservoir Manag.* 22 213–222. 10.1080/07438140609353898

[B43] Kurahashi M. Fukunaga Y. Sakiyama Y. Harayama S. Yokota A. (2009). *Iamia majanohamensis* gen. nov., sp. nov., an actinobacterium isolated from sea cucumber *Holothuria edulis*, and proposal of Iamiaceae fam. nov. *Int. J. Syst. Evol. Microbiol.* 59 869–873. 10.1099/ijs.0.005611-0 19329622

[B44] Lane D. J. (1991). “16S/23S rRNA sequencing,” in *Nucleic Acid Techniques in Bacterial Systematics*, eds Stackebrandt E. Goodfellow M. (New York, NY: John Wiley & Sons), 115–175.

[B45] Lee J. Kwon K. K. Lim S. I. Song J. Choi A. R. Yang S. H. (2019). Isolation, cultivation, and genome analysis of proteorhodopsin-containing SAR116-clade strain Candidatus Puniceispirillum marinum IMCC1322. *J. Microbiol.* 57 676–687. 10.1007/s12275-019-9001-2 31201724

[B46] Lewis W. H. Tahon G. Geesink P. Sousa D. Z. Ettema T. J. G. (2021). Innovations to culturing the uncultured microbial majority. *Nat. Rev. Microbiol.* 19 225–240. 10.1038/s41579-020-00458-8 33093661

[B47] Lindstrom E. S. Kamst-Van Agterveld M. P. Zwart G. (2005). Distribution of typical freshwater bacterial groups is associated with pH, temperature, and lake water retention time. *Appl. Environ. Microbiol.* 71 8201–8206. 10.1128/AEM.71.12.8201-8206.2005 16332803 PMC1317352

[B48] Liu Z. T. Jiao J. Y. Liu L. Li M. M. Ming Y. Z. Song J. L. (2021). *Rhabdothermincola sediminis* gen. nov., sp. nov., a new actinobacterium isolated from hot spring sediment, and emended description of the family Iamiaceae. *Int. J. Syst. Evol. Microbiol.* 71:004760. 10.1099/ijsem.0.004760 33739250

[B49] Ludwig W. Strunk O. Westram R. Richter L. Meier H. Yadhukumar (2004). ARB: a software environment for sequence data. *Nucleic Acids Res.* 32 1363–1371. 10.1093/nar/gkh293 14985472 PMC390282

[B50] Martinez-Garcia M. Brazel D. M. Swan B. K. Arnosti C. Chain P. S. G. Reitenga K. G. (2012a). Capturing single cell genomes of active polysaccharide degraders: an unexpected contribution of Verrucomicrobia. *PLoS One* 7:e35314. 10.1371/journal.pone.0035314 22536372 PMC3335022

[B51] Martinez-Garcia M. Swan B. K. Poulton N. J. Gomez M. L. Masland D. Sieracki M. E. (2012b). High-throughput single-cell sequencing identifies photoheterotrophs and chemoautotrophs in freshwater bacterioplankton. *ISME J.* 6 113–123. 10.1038/ismej.2011.84 21716306 PMC3246240

[B52] Matsumoto A. Kasai H. Matsuo Y. Omura S. Shizuri Y. Takahashi Y. (2009). *Ilumatobacter fluminis* gen. nov., sp. nov., a novel actinobacterium isolated from the sediment of an estuary. *J. Gen. Appl. Microbiol.* 55 201–205. 10.2323/jgam.55.201 19590147

[B53] Moon K. Kang I. Kim S. Cho J. C. Kim S. J. (2015). Complete genome sequence of bacteriophage P26218 infecting *Rhodoferax* sp. strain IMCC26218. *Stand. Genomic Sci.* 10:111. 10.1186/S40793-015-0090-1 26605005 PMC4657236

[B54] Moon K. Kang I. Kim S. Kim S. J. Cho J. C. (2017). Genome characteristics and environmental distribution of the first phage that infects the LD28 clade, a freshwater methylotrophic bacterial group. *Environ. Microbiol.* 19 4714–4727. 10.1111/1462-2920.13936 28925542

[B55] Moon K. Kang I. Kim S. Kim S.-J. Cho J.-C. (2018). Genomic and ecological study of two distinctive freshwater bacteriophages infecting a Comamonadaceae bacterium. *Sci. Rep.* 8:7989. 10.1038/s41598-018-26363-y 29789681 PMC5964084

[B56] Nakai R. Abe T. Baba T. Imura S. Kagoshima H. Kanda H. (2012). Microflorae of aquatic moss pillars in a freshwater lake, East Antarctica, based on fatty acid and 16S rRNA gene analyses. *Polar Biol.* 35 425–433. 10.1007/s00300-011-1090-2

[B57] Nam G. G. Joung Y. Park M. Kim S. Jeon H. T. Cho J. C. (2017). *Flavobacterium soyangense* sp nov., a psychrotolerant bacterium, isolated from an oligotrophic freshwater lake. *Int. J. Syst. Evol. Microbiol.* 67 2440–2445. 10.1099/ijsem.0.001987 28742006

[B58] Neuenschwander S. M. Ghai R. Pernthaler J. Salcher M. M. (2018). Microdiversification in genome-streamlined ubiquitous freshwater Actinobacteria. *ISME J.* 12 185–198. 10.1038/ismej.2017.156 29027997 PMC5739012

[B59] Newton R. J. Jones S. E. Eiler A. McMahon K. D. Bertilsson S. (2011). A guide to the natural history of freshwater lake bacteria. *Microbiol. Mol. Biol. Rev.* 75 14–49. 10.1128/MMBR.00028-10 21372319 PMC3063352

[B60] Okazaki Y. Fujinaga S. Salcher M. M. Callieri C. Tanaka A. Kohzu A. (2021). Microdiversity and phylogeographic diversification of bacterioplankton in pelagic freshwater systems revealed through long-read amplicon sequencing. *Microbiome* 9:24. 10.1186/s40168-020-00974-y 33482922 PMC7825169

[B61] Park M. Nam G. G. Kim S. Jeon H. T. Joung Y. Cho J. C. (2017). *Flavobacterium chuncheonense* sp. nov. and *Flavobacterium luteum* sp. nov., isolated from a freshwater lake. *Int. J. Syst. Evol. Microbiol.* 67 4409–4415. 10.1099/ijsem.0.002304 28920853

[B62] Philosof A. Sabehi G. Beja O. (2009). Comparative analyses of actinobacterial genomic fragments from Lake Kinneret. *Environ. Microbiol.* 11 3189–3200. 10.1111/j.1462-2920.2009.02024.x 19678830

[B63] Pruesse E. Peplies J. Glockner F. O. (2012). SINA: accurate high-throughput multiple sequence alignment of ribosomal RNA genes. *Bioinformatics* 28 1823–1829. 10.1093/bioinformatics/bts252 22556368 PMC3389763

[B64] Quast C. Pruesse E. Yilmaz P. Gerken J. Schweer T. Yarza P. (2013). The SILVA ribosomal RNA gene database project: improved data processing and web-based tools. *Nucleic Acids Res.* 41 D590–D596. 10.1093/nar/gks1219 23193283 PMC3531112

[B65] Rappe M. S. Connon S. A. Vergin K. L. Giovannoni S. J. (2002). Cultivation of the ubiquitous SAR11 marine bacterioplankton clade. *Nature* 418 630–633. 10.1038/nature00917 12167859

[B66] Reasoner D. J. Geldreich E. E. (1985). A new medium for the enumeration and subculture of bacteria from potable water. *Appl. Environ. Microbiol.* 49 1–7. 10.1128/AEM.49.1.1-7.1985 3883894 PMC238333

[B67] Rinke C. Schwientek P. Sczyrba A. Ivanova N. N. Anderson I. J. Cheng J. F. (2013). Insights into the phylogeny and coding potential of microbial dark matter. *Nature* 499 431–437. 10.1038/nature12352 23851394

[B68] Rohwer R. R. Hamilton J. J. Newton R. J. McMahon K. D. (2018). TaxAss: leveraging a custom freshwater database achieves fine-scale taxonomic resolution. *mSphere* 3:e00327-18. 10.1128/mSphere.00327-18 30185512 PMC6126143

[B69] Salcher M. M. Neuenschwander S. M. Posch T. Pernthaler J. (2015). The ecology of pelagic freshwater methylotrophs assessed by a high-resolution monitoring and isolation campaign. *ISME J.* 9 2442–2453. 10.1038/ismej.2015.55 25942006 PMC4611508

[B70] Salcher M. M. Schaefle D. Kaspar M. Neuenschwander S. M. Ghai R. (2019). Evolution in action: habitat transition from sediment to the pelagial leads to genome streamlining in Methylophilaceae. *ISME J.* 13 2764–2777. 10.1038/s41396-019-0471-3 31292537 PMC6794327

[B71] Sangwan N. Zarraonaindia I. Hampton-Marcell J. T. Ssegane H. Eshoo T. W. Rijal G. (2016). Differential functional constraints cause strain-level endemism in Polynucleobacter populations. *mSystems* 1:e00003-16. 10.1128/mSystems.00003-16 27822527 PMC5069759

[B72] Schloss P. D. Gevers D. Westcott S. L. (2011). Reducing the effects of PCR amplification and sequencing artifacts on 16S rRNA-based studies. *PLoS One* 6:e27310. 10.1371/journal.pone.0027310 22194782 PMC3237409

[B73] Schloss P. D. Westcott S. L. Ryabin T. Hall J. R. Hartmann M. Hollister E. B. (2009). Introducing mothur: open-source, platform-independent, community-supported software for describing and comparing microbial communities. *Appl. Environ. Microbiol.* 75 7537–7541. 10.1128/Aem.01541-09 19801464 PMC2786419

[B74] Shabarova T. Kasalický V. Šimek K. Nedoma J. Znachor P. Posch T. (2017). Distribution and ecological preferences of the freshwater lineage LimA (genus *Limnohabitans*) revealed by a new double hybridization approach. *Environ. Microbiol.* 19 1296–1309. 10.1111/1462-2920.13663 28063252

[B75] Shah V. Chang B. X. Morris R. M. (2017). Cultivation of a chemoautotroph from the SUP05 clade of marine bacteria that produces nitrite and consumes ammonium. *ISME J.* 11 263–271. 10.1038/ismej.2016.87 27434424 PMC5315479

[B76] Song J. Oh H. M. Cho J. C. (2009). Improved culturability of SAR11 strains in dilution-to-extinction culturing from the East Sea, West Pacific Ocean. *FEMS Microbiol. Lett.* 295 141–147. 10.1111/j.1574-6968.2009.01623.x 19459973

[B77] Spietz R. L. Lundeen R. A. Zhao X. W. Nicastro D. Ingalls A. E. Morris R. M. (2019). Heterotrophic carbon metabolism and energy acquisition in Candidatus Thioglobus singularis strain PS1, a member of the SUP05 clade of marine Gammaproteobacteria. *Environ. Microbiol.* 21 2391–2401. 10.1111/1462-2920.14623 30951247

[B78] Stein L. Y. Jones G. Alexander B. Elmund K. Wright-Jones C. Nealson K. H. (2002). Intriguing microbial diversity associated with metal-rich particles from a freshwater reservoir. *FEMS Microbiol. Ecol.* 42 431–440. 10.1111/j.1574-6941.2002.tb01032.x 19709302

[B79] Stingl U. Desiderio R. A. Cho J. C. Vergin K. L. Giovannoni S. J. (2007). The SAR92 clade: an abundant coastal clade of culturable marine bacteria possessing proteorhodopsin. *Appl. Environ. Microbiol.* 73 2290–2296. 10.1128/Aem.02559-06 17293499 PMC1855678

[B80] Warnecke F. Amann R. Pernthaler J. (2004). Actinobacterial 16S rRNA genes from freshwater habitats cluster in four distinct lineages. *Environ. Microbiol.* 6 242–253. 10.1111/j.1462-2920.2004.00561.x 14871208

[B81] Williams T. A. Embley T. M. (2014). Archaeal “dark matter” and the origin of eukaryotes. *Genome Biol. Evol.* 6 474–481. 10.1093/gbe/evu031 24532674 PMC3971582

[B82] Williams T. J. Wilkins D. Long E. Evans F. Demaere M. Z. Raftery M. J. (2013). The role of planktonic *Flavobacteria* in processing algal organic matter in coastal East Antarctica revealed using metagenomics and metaproteomics. *Environ. Microbiol.* 15 1302–1317. 10.1111/1462-2920.12017 23126454

[B83] Wu Q. L. Hahn M. W. (2006a). Differences in structure and dynamics of *Polynucleobacter* communities in a temperate and a subtropical lake, revealed at three phylogenetic levels. *FEMS Microbiol. Ecol.* 57 67–79. 10.1111/j.1574-6941.2006.00105.x 16819951

[B84] Wu Q. L. Hahn M. W. (2006b). High predictability of the seasonal dynamics of a species-like *Polynucleobacter* population in a freshwater lake. *Environ. Microbiol.* 8 1660–1666. 10.1111/j.1462-2920.2006.01049.x 16913925

[B85] Zhang W. W. Li F. Nie L. (2010). Integrating multiple ‘omics’ analysis for microbial biology: application and methodologies. *Microbiology* 156 287–301. 10.1099/mic.0.034793-0 19910409

[B86] Zwart G. Crump B. C. Agterveld M. P. K. V. Hagen F. Han S. K. (2002). Typical freshwater bacteria: an analysis of available 16S rRNA gene sequences from plankton of lakes and rivers. *Aquat. Microb. Ecol.* 28 141–155. 10.3354/Ame028141

