# Supplementary material for: Cultivation of Dominant Freshwater Bacterioplankton Lineages Using a High-Throughput Dilution-to-Extinction Culturing Approach Over a 1-Year Period
Source: Front Microbiol. 2021 Jul 27;12:700637. doi: 10.3389/fmicb.2021.700637 (PMC8353197; doi:10.3389/fmicb.2021.700637)
Supplement: Supplementary file 1 [file Data_Sheet_1.PDF]

Supplementary Material

1 Supplementary Data

Separate MS-Excel file supplied.

Supplementary Data. List of all HTC isolates obtained in this study. Each strain is shown with culture ID, isolation information, taxonomic position, and GenBank accession number in the Excel file.

2 Supplementary Figures and Tables

2.1 Supplementary Figures

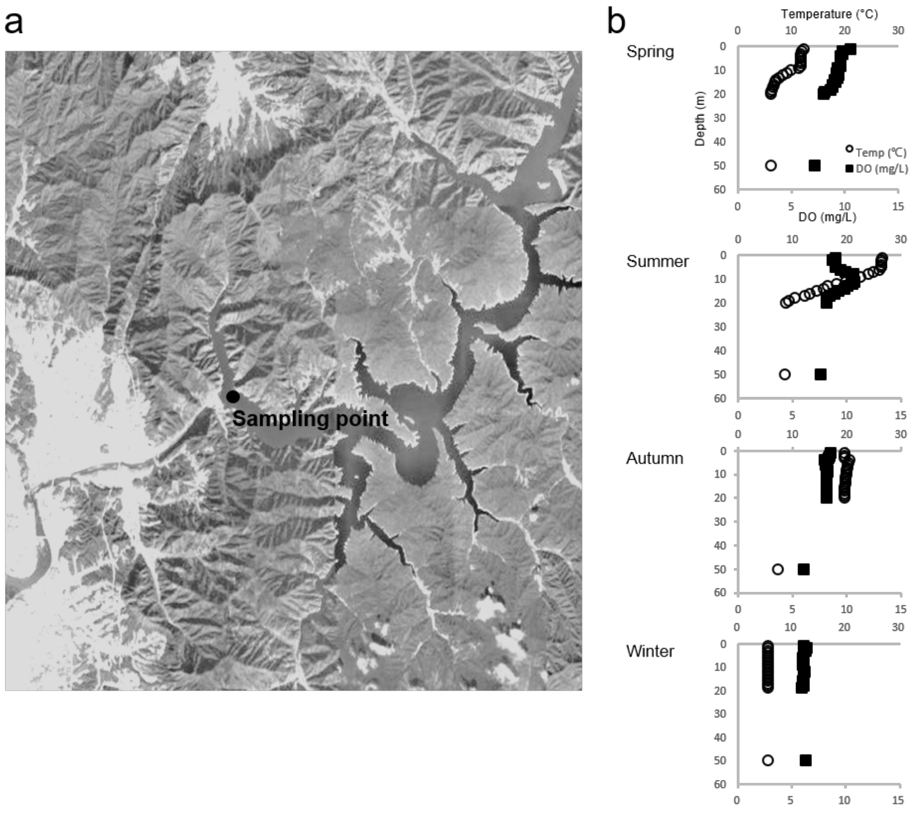

Supplementary Figure 1. Location of sampling station (a) and vertical profiles of temperature and dissolved oxygen in spring, summer, autumn, and winter (b).

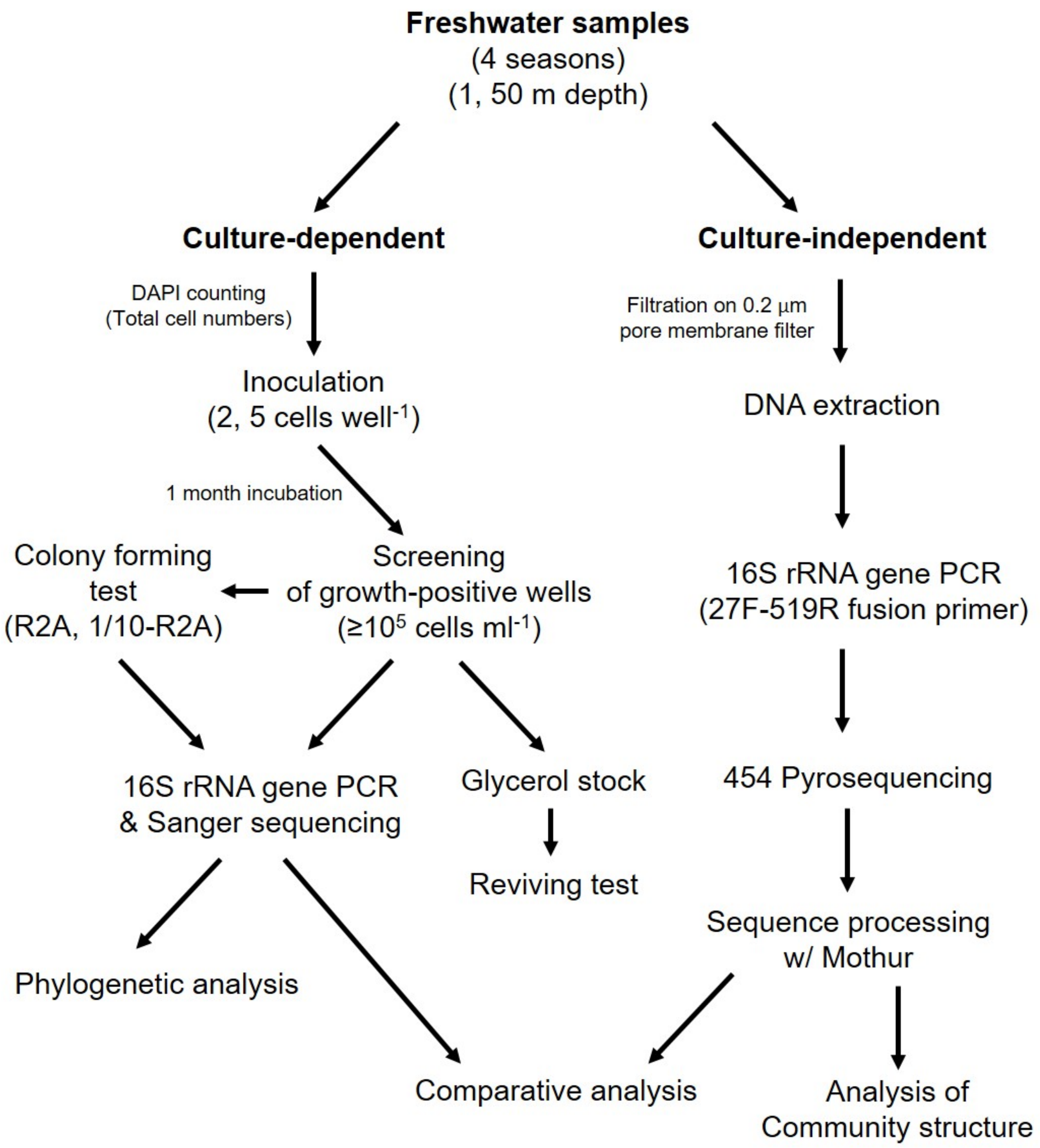

Supplementary Figure 2. Experimental scheme applied in this study.

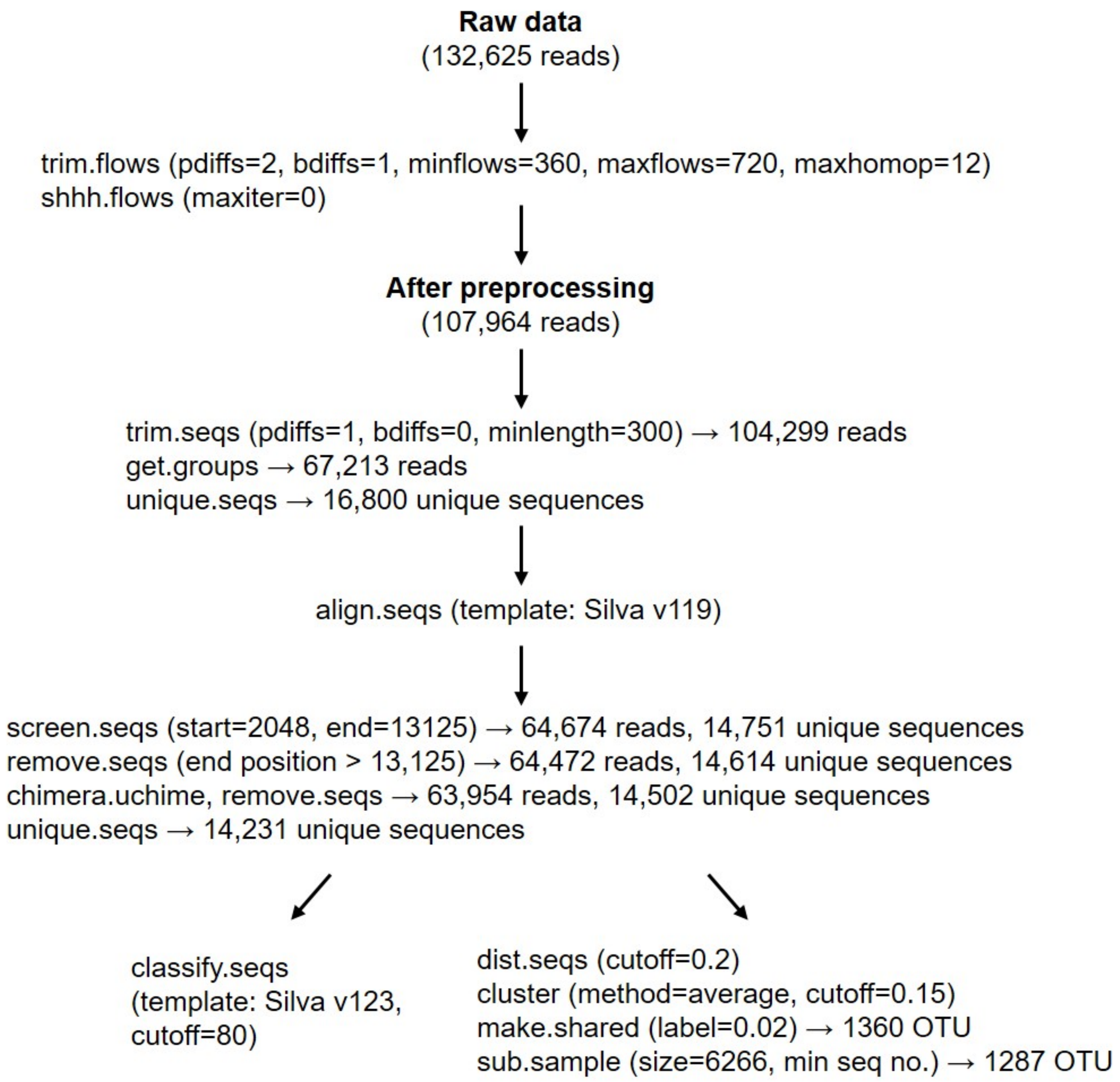

Supplementary Figure 3. Workflow for the 16S rRNA gene pyrosequencing analysis using the Mothur software.

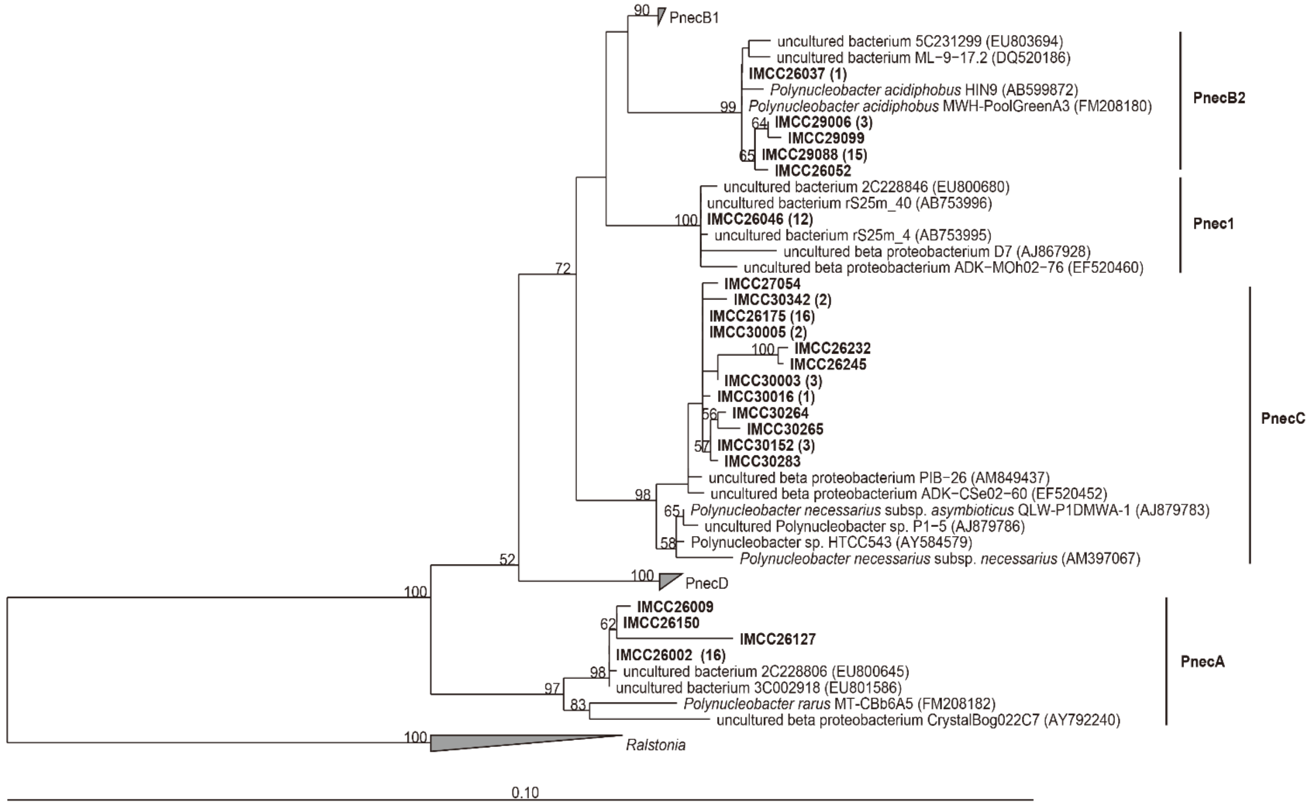

Supplementary Figure 4. Maximum-likelihood phylogenetic tree based on 16S rRNA gene sequences showing the positions of the HTC isolates within the genus *Polynucleobacter*. The HTC isolates are prefixed with the "IMCC" abbreviation (Inha Microbe Culture Collection) and shown in boldface. HTC isolates sharing identical 16S rRNA gene sequences are indicated by one representative strain and the numbers of the other isolates sharing the same sequences are indicated in parentheses. The Pnec1 lineage was named in this study. Bootstrap supporting values are shown at the nodes.

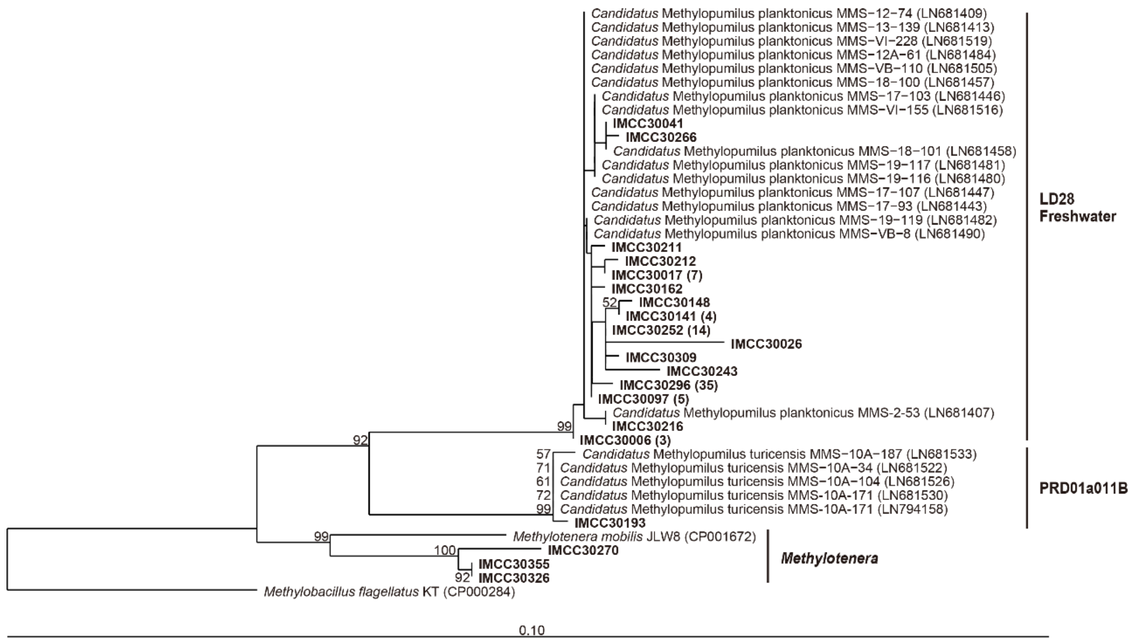

Supplementary Figure 5. Maximum-likelihood phylogenetic tree based on 16S rRNA gene sequences showing the positions of the HTC isolates within the family *Methylophilaceae*. The HTC isolates are prefixed with the "IMCC" abbreviation (Inha Microbe Culture Collection) and shown in boldface. HTC isolates sharing identical 16S rRNA gene sequences are indicated by one representative strain and the numbers of the other isolates sharing the same sequences are indicated in parentheses. Bootstrap supporting values are shown at the nodes.

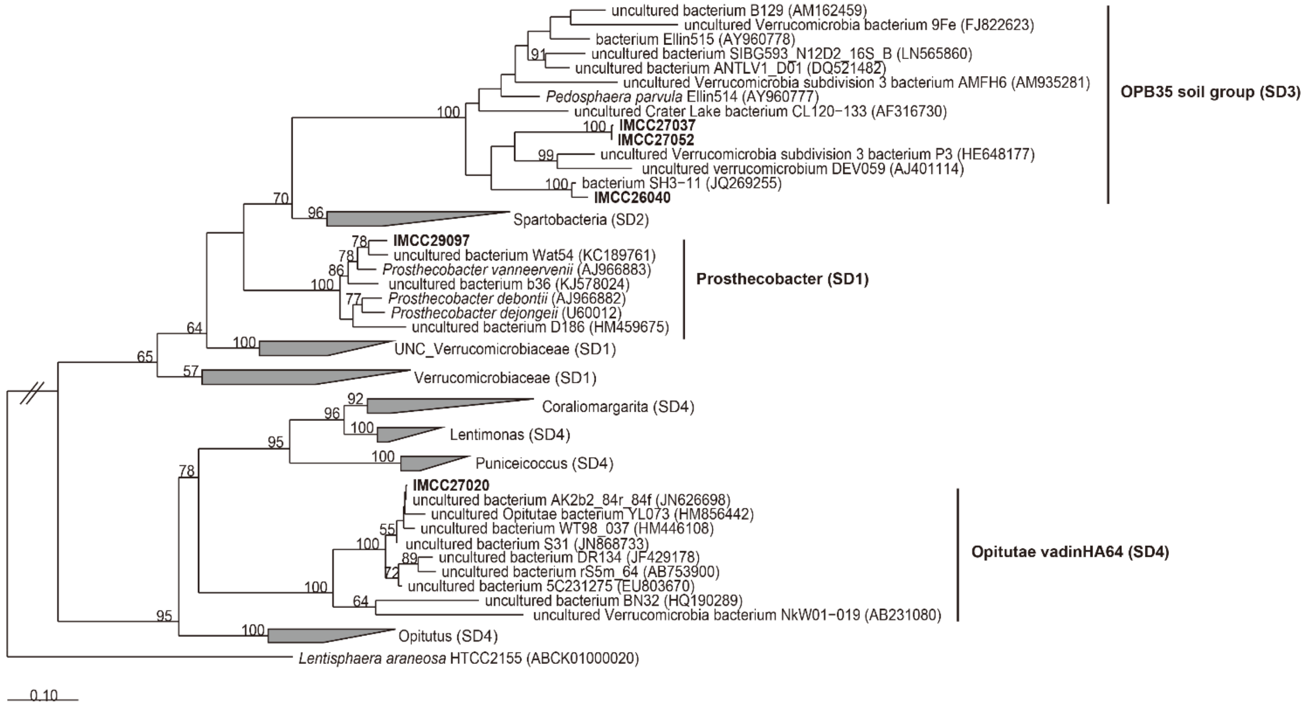

Supplementary Figure 6. Maximum-likelihood phylogenetic tree based on 16S rRNA gene sequences showing the positions of the HTC isolates within the phylum *Verrucomicrobia*. The HTC isolates are prefixed with the "IMCC" abbreviation (Inha Microbe Culture Collection) and shown in boldface. HTC isolates sharing identical 16S rRNA gene sequences are indicated by one representative strain and the numbers of the other isolates sharing the same sequences are indicated in parentheses. Bootstrap supporting values are shown at the nodes.

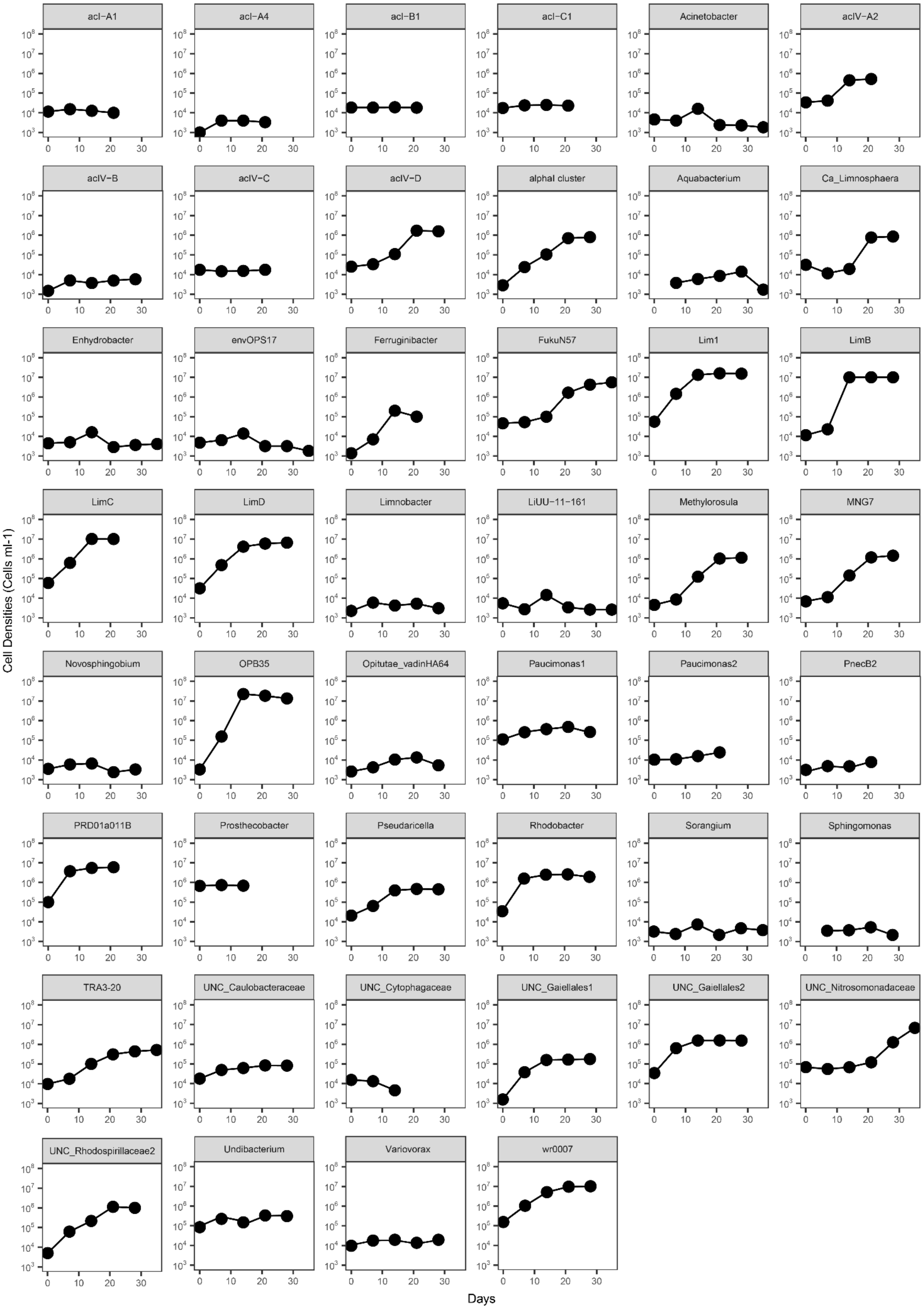

Supplementary Figure 7. Growth curves of 46 HTC isolates belonging to 46 phylogenetic groups, revived from cryopreservation.

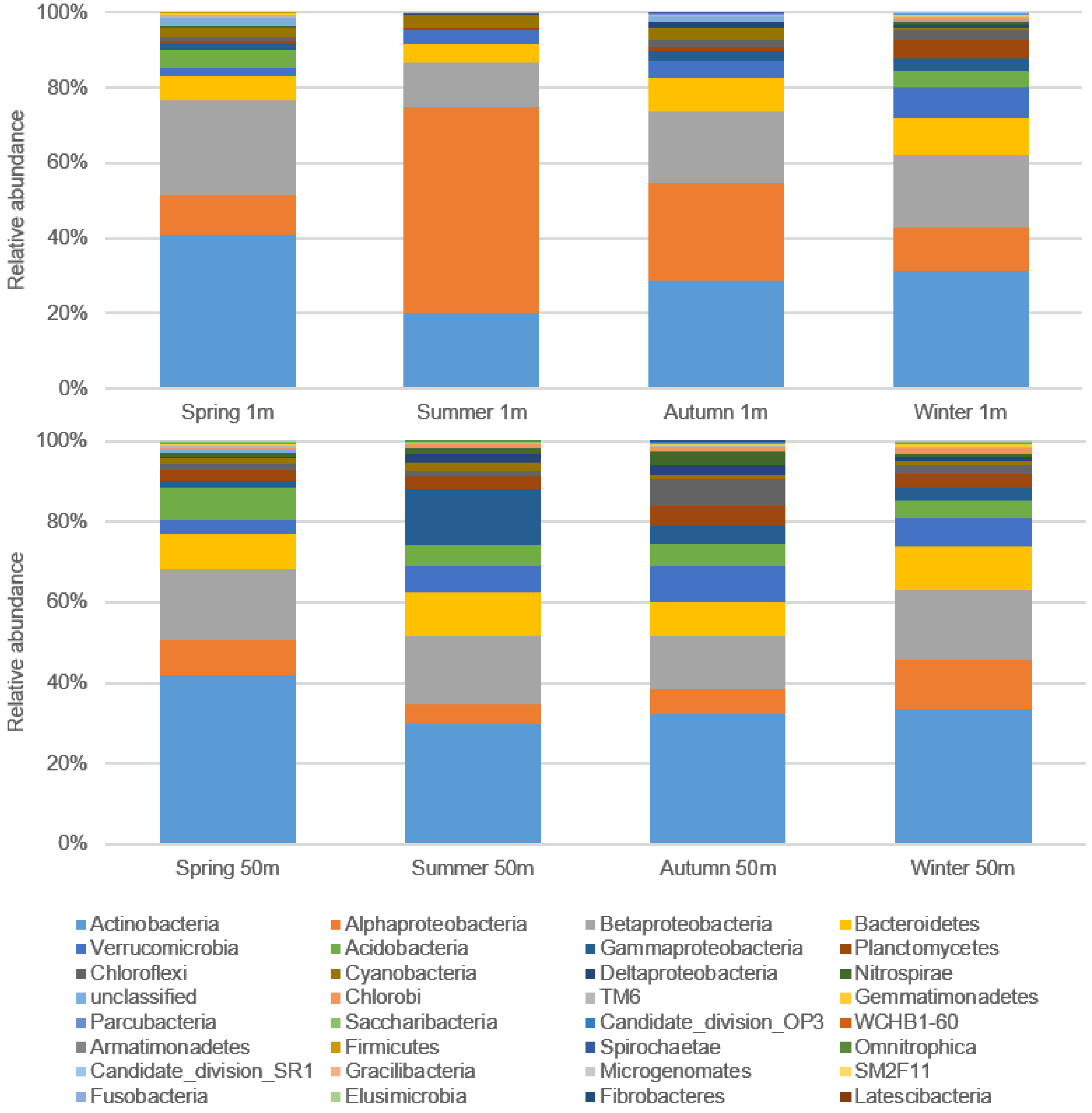

Supplementary Figure 8. Relative abundance of phyla (classes for *Proteobacteria*) in the bacterial community of eight Lake Soyang samples obtained by pyrosequencing.

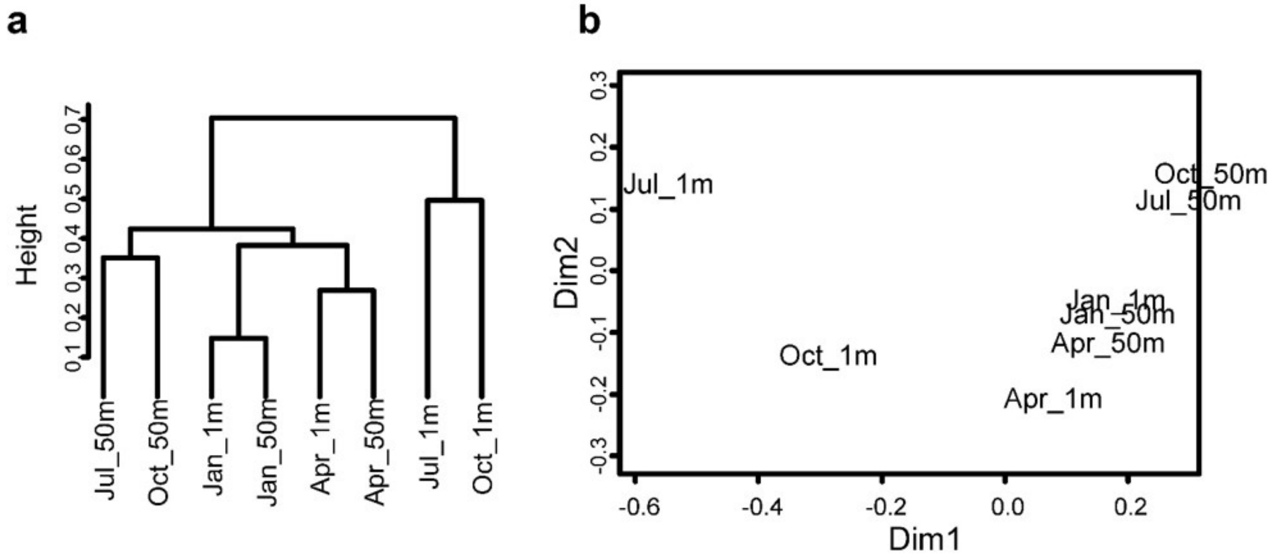

Supplementary Figure 9. Comparison of the bacterial community of Lake Soyang, analyzed by hierarchical clustering with UPGMA (a) and PCoA (b) using Bray-Curtis distance.

2.2 Supplementary Tables

Supplementary Table 1. Physicochemical characteristics of the lake water samples used for HTC experiments and tag-pyrosequencing.

	Spring		Summer		Autumn		Winter	
	1 m	50 m	1 m	50 m	1 m	50 m	1 m	50 m
Sampling site (GPS)	37°56'49.2" N, 127°48'57.6" E							
Temperature (°C)	12.3	6.1	26.5	8.6	19.5	7.3	5.6	5.4
Salinity	0.03	0.03	0.03	0.03	0	0.04	0.04	0.04
Conductivity (us/㎝)	68	71	74	73	79	72	80	80
pH	7.9	7.2	8.4	7.1	7.1	6.7	6.9	6.9
DO (㎎/L)	10.5	7.1	9.0	7.6	8.5	6.1	6.1	6.2
Nitrite (mg/L NO_2^--N)	0.008	0.003	0.015	0.004	0.007	0.004	0.006	0.004
Nitrate (mg/L NO_3^--N)	1.4	1.4	1.3	1.3	1.5	1.5	1.2	1.2
Ammonium (mg/L NH_3-N)	0.01	0.01	0	0	0.01	0	0	0
Phosphate (mg/L PO_4^{3-})	0.02	0	0.01	0.01	0.01	0	0.01	0
Silicate (mg/L SiO_2)	2.0	2.0	1.7	2.5	2.4	2.7	2.6	2.3
Chlorophyll *a* (mg/L)	1.74	0.92	1.00	0.27	7.88	0.93	2.93	1.96
Total prokaryotic number (10^6 cells/ml)	1.02	0.77	2.87	1.47	2.63	0.99	1.48	1.44
Total dissolved nitrogen (mg/L)	1.47	1.52	1.54	1.51	1.67	1.53	1.58	1.54
Dissolved organic carbon (mg/L)	2.34	2.63	3.03	2.98	2.74	2.15	1.96	1.91

Supplementary Table 2. Media composition used for HTC experiments in this study.

Components	Compound(s)	Final concentration
Ammonium	NH_4Cl	10 μM
Phosphate	KH_2PO_4	10 μM
Trace metals	$FeCl_3 \cdot 6H_2O$	117 nM
	$MnCl_2 \cdot 4H_2O$	9 nM
	$ZnSO_4 \cdot 7H_2O$	800 pM
	$CoCl_2 \cdot 6H_2O$	500 pM
	$Na_2MoO_4 \cdot 2H_2O$	300 pM
	Na_2SeO_3	1 nM
	$NiCl_2 \cdot 6H_2O$	1 nM
Vitamin mixture	Thiamine·HCl	59 nM
	Niacin	81 nM
	Ca-Pantothenate	84 nM
	Pyridoxine	59 nM
	Biotin	409 pM
	Folic acid	453 pM
	Vitamin B12	70 pM
	Myo-inositol	555 nM
	p-Aminobenzoic Acid	7 nM
Carbon mixture	Pyruvate	50 μM
	D-Glucose	5 μM
	N-Acetyl-D-glucosamine	5 μM
	D-Ribose	5 μM
	Methyl alcohol	5 μM
20 proteinogenic amino acid mixture	Each amino acid	100 nM, each

Supplementary Table 3. The numbers of HTC isolates and colony-forming strains and revivability from glycerol stocks for each phylogenetic group. Y, revived; N, non-revived; -, non-tested. The number in parenthesis represents IMCC strain ID.

Phylogenetic group	isolate	# colony	Revivability (IMCC)
Actinobacteria			
Acidimicrobiales			
acIV-A2	6	0	Y (27063)
acIV-B	1	0	N
acIV-C	1	0	N
acIV-D	9	0	Y (26085)
Ca. Limnosphaera	2	0	Y (26207)
Frankiales			
acI-A1	2	0	N
acI-A4	1	0	N
acI-B1	1	0	N
acI-C1	1	0	N
Gaiellales			
uncultured group 1	3	0	Y (30188)
uncultured group 2	22	0	Y (30110)
Alphaproteobacteria			
Caulobacterales			
Caulobacter	4	4	-
Brevundimonas	1	1	-
uncultured Caulobacteraceae	1	0	N
Phenylobacterium	10	10	-
Rhizobiales			
A0839	2	1	-
Alpha1 cluster	1	0	Y (30301)
Methylorosula	1	0	Y (30094)
Bradyrhizobium_Afpia	2	1	-
FukuN57	6	0	Y (29117)
MNG7	1	0	Y (30267)
Rhodobacterales			
Rhodobacter	6	0	Y (30069)
Defluvvimonas	1	1	-
Hirschia	9	7	-
Unc. Hyphomonadaceae	1	1	-
Rhodospirillales			
Uncultured Rhodospirillales1	1	1	-
Uncultured Rhodospirillales2	1	0	Y (30107)
Ferrovibrio	3	3	-
Wr0007	1	0	Y (30051)
Sphingomonadales			
GOBB3-C201	7	4	-
ovosphingobium	1	0	N
Sphingomonas	1	0	N
Sandarakinorhabdus	1	1	-
Gammaproteobacteria			
Acinetobacter	1	0	N
Enhydrobacter	1	0	N
Arenimonas	1	1	-

	No. isolate	Col	Revivability (IMCC)
Betaproteobacteria			
Burkholderiales			
TRA3-20	8	0	Y (27049)
PnecA	20	5	-
PnecB2	24	0	N
PnecC	39	36	-
Pnec	13	10	-
LimB	80	0	Y (26090)
LimC	1	0	Y (27035)
LimD	36	0	Y (27031)
Lim1	4	0	Y (30102, 30221)
Lim2	52	3	-
Variovorax	1	0	N
Rhodoferax_Albidiferax	53	5	-
Polaromonas	9	2	-
Aquabacterium	1	0	N
Rhizobacter	1	1	-
Limnobacter	2	0	N
Undibacterium	1	0	N
Paucimonas1	5	0	N
Paucimonas2	2	0	N
Methylophilaceae			
LD28	84	2	-
Methylotenera	3	2	-
PRD01a011B	1	0	Y (30193)
Nitrosomonadales			
Uncultured Nitrosomonadaceae	3	0	Y (29147,30076,29158)
MWH-UniP1	1	1	-
GKS98	5	1	-
Deltaproteobacteria			
Myxococcales			
Sorangium	1	0	N
Verrucomicrobia			
OPB35 soil group	3	0	Y (26040)
vadinHA64	1	0	N
Verrucomicrobiales			
Prosthecobacter	1	0	N
Bacteroidetes			
UNC Cytophagaceae	1	0	N
Flavobacterium	27	15	-
Pseudarcicella	4	0	Y (26057)
LiUU-11-161	1	0	N
env.OPS_17	1	0	N
Ferruginibacter	2	0	Y (26056)

Supplementary Table 4. Number of pyrosequencing reads consistent with HTC isolates, as determined by BLASTn.

	Spring		Summer		Autumn		Winter	
	1 m	50 m	1 m	50 m	1 m	50 m	1 m	50 m
Total read number	7125	6266	8384	7072	7372	6840	6708	7489
97% similarity								
Matched read number	2838	1603	1711	1680	2297	1120	2015	2047
% of match	39.8	25.6	20.4	23.8	31.2	16.4	30	27.3
98.7% similarity								
Matched read number	2703	1477	1660	1569	2122	956	1734	1802
% of match	37.9	20.7	23.3	22	29.8	13.4	24.3	25.3
